# Personalized Immunotherapy for T Cell Lymphomas: From Immune Escape to Precision Therapeutics

**DOI:** 10.3390/jpm15110560

**Published:** 2025-11-18

**Authors:** Joshua M. L. Casan, Xiao Jing Ong, Carrie van der Weyden, Costas K. Yannakou, Joe Zhu, Criselle D’Souza, Paul Neeson, H. Miles Prince

**Affiliations:** 1 The Sir Peter MacCallum Department of Oncology, Faculty of Medicine, Dentistry and Health Science, University of Melbourne, Melbourne, VIC 3010, Australia; xiaojing.ong@petermac.org (X.J.O.); carrie.vanderweyden@petermac.org (C.v.d.W.); joe.zhu@petermac.org (J.Z.); criselle.dsouza@petermac.org (C.D.); paul.neeson@petermac.org (P.N.); miles.prince@petermac.org (H.M.P.); 2 Department of Clinical Hematology, Peter MacCallum Cancer Centre, Melbourne, VIC 3000, Australia; 3 Cancer Immunology Program, Peter MacCallum Cancer Centre, Melbourne, VIC 3000, Australia; 4 Epworth HealthCare, Melbourne, VIC 3121, Australia; 5 Department of Clinical Pathology, Faculty of Medicine, Dentistry and Health Science, University of Melbourne, Melbourne, VIC 3010, Australia

**Keywords:** T cell lymphoma, peripheral T-cell lymphomas (PTCL), immunotherapy, tumor microenvironment (TME), immune checkpoint inhibitors, CAR-T cell therapy, biomarkers

## Abstract

Despite recent progress in lymphoma immunotherapy, outcomes for patients with peripheral T cell lymphomas (PTCLs) remain poor. The challenge of PTCLs reflects the profound biological heterogeneity and relative rarity of this disease group and its resistance to conventional chemotherapy, as well as the formidable challenge of generating definitive clinical evidence. However, deepening insight into the immunogenomic and microenvironmental basis of PTCL has revealed diverse mechanisms of immune escape, spanning defects in antigen presentation, apoptotic signaling, adhesion, and extensive tumor microenvironmental remodeling. These vulnerabilities provide a sound rationale for novel immunotherapeutic strategies—checkpoint inhibitors, CAR-T and NK cell platforms, bispecific antibodies, oncolytic viruses, and immunomodulatory agents. Early studies show encouraging but inconsistent activity, and the variability in response highlights the urgent need for biomarker-driven stratification to deliver personalized approaches and clinically meaningful efficacy. This review synthesizes the current literature on the immune dysregulation of PTCLs, as well as advances in PTCL immunotherapy, outlining the biological rationale underpinning these approaches. We discuss approaches to molecular, transcriptomic, and microenvironmental profiling with circulating biomarkers that could enable adaptive trial designs and personalized treatment strategies. Together, these developments chart a path away from empiricism and toward precision therapy in PTCLs.

## 1. Introduction

Peripheral T cell lymphomas (PTCLs) are a biologically and clinically heterogeneous group of malignancies that remain among the most challenging lymphomas to treat. PTCLs collectively account for approximately 10–15% of non-Hodgkin lymphomas worldwide but remain rare in Western populations, where they comprise less than 5% of cases [1]. Their incidence and subtype distribution show marked geographic variation, reflecting distinct pathogenetic and environmental influences [1]. Within this broad category, there are more than 30 distinct subtypes, with highly variable clinicopathological presentation and outcomes [2]. Their rarity and diversity have historically meant treatment strategies are extrapolated from B cell lymphoma, despite fundamental biological differences. Consequently, conventional chemotherapy regimens, while still used frontline, often yield only transient responses, and outcomes for relapsed or refractory disease remain abysmal [2,3]. Increasing evidence from molecular and immunogenomic profiling has elucidated the profound immune dysregulation underpinning PTCL biology, including defects in antigen presentation, deregulated apoptotic pathways, altered adhesion signaling, and remodeling of the tumor microenvironment. These discoveries confirm what clinical practice has repeatedly suggested: that PTCL pathobiology involves many distinct immune escape mechanisms that demand tailored therapeutic approaches.

The rapid evolution of novel immunotherapeutics has generated unprecedented opportunities to exploit such vulnerabilities. Checkpoint inhibitors, engineered cellular therapies, such as Chimeric Antigen Receptor (CAR)-T and CAR-NK cells, bispecific antibodies, oncolytic viruses, and immunomodulatory drugs are promising treatments that continue to evolve and transform outcomes for cancer patients. However, the variable efficacy of these agents across different cancers highlights the limitations of “one-size-fits-all” therapy and the urgency of developing biomarker-driven patient selection frameworks. Current personalized approaches are still in their infancy, often limited to subgroup analyses or exploratory biomarker studies, but they represent the first steps towards implementing true precision medicine in cancer generally and PTCL specifically. By integrating genomic, immunophenotypic, and microenvironmental data, it may be possible to stratify patients more effectively, match them to the therapies most likely to succeed, and avoid futile or harmful interventions. This review explores the current state of PTCL immunotherapy, the biological rationale underpinning these strategies, and the future directions that may ultimately deliver durable and personalized disease control. The focus of this article is on PTCLs, though relevant data from cutaneous T cell lymphoma (CTCL) will be discussed where relevant and important realms of overlap will be acknowledged.

## 2. Immunobiology of Peripheral T Cell Lymphomas (PTCLs)

T cell lymphomas (TCLs) represent a heterogeneous group of malignancies, arising from either innate lymphoid cells, such as natural killer (NK) and gamma-delta (ɣδ) T cells, or from CD8+ and, more commonly, CD4+ T-helper cells of the adaptive immune compartment. Multiple tumor-intrinsic mechanisms have been shown to contribute to T cell lymphomagenesis, including the acquisition of genomic alterations in the JAK-STAT signaling pathways, T cell receptor (TCR), T cell activation-related genes (CD28, CD40, PI3K-AKT, AP-1), and, in some subtypes, the RHOA pathway and epigenetic modifier genes. Multiple reviews have described these intrinsic defects in detail [4,5].

**Figure 1:**
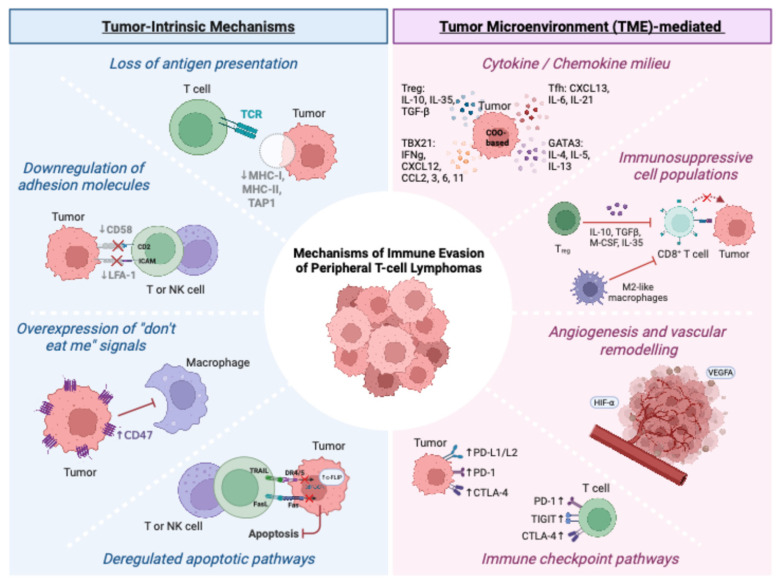
Schematic representation of tumor-intrinsic and tumor-extrinsic immune evasion pathways in peripheral T cell lymphomas. Malignant T cells evade immune surveillance through impaired antigen presentation (HLA/TAP loss), adhesion molecule downregulation (CD58/LFA-1), upregulation of anti-phagocytic signals (CD47), and resistance to apoptosis (Fas/TRAIL pathways). In parallel, the tumor microenvironment is reshaped into an immunosuppressive niche, mediated by regulatory T cells, M2-like macrophages, inhibitory cytokines, and angiogenesis. Together, these mechanisms foster immune escape and therapeutic resistance.

The interaction between TCL cells and their tumor microenvironment (TME) contributes another layer of complexity in this lymphomagenesis process. The immune system plays a crucial role in tumor immune surveillance, eliminating transformed cells under normal circumstances [6]. However, TCL cells can evolve and acquire tumor-intrinsic changes, such as downregulation of antigen presentation and adhesion molecules, upregulation of “don’t eat me” signals, and dysregulated apoptotic signals, that allow them to escape immune destruction. In parallel, the TME can be remodeled into a pro-tumorigenic, immune-suppressive niche through the recruitment of immunosuppressive cells, the secretion of inhibitory cytokines, the upregulation of immune checkpoint, and vascular remodeling, thereby further supporting tumor growth and survival [7,8]. This section will provide an in-depth insight into these processes within the context of TCL (Figure 1).

Understanding the escape from tumor immune surveillance is not only critical for elucidating disease biology but also for identifying patient-specific vulnerabilities that can be exploited by personalized immunotherapeutic strategies aimed at reprogramming the tumor–immune interface. Emerging modalities such as checkpoint inhibitors, CAR-T cells, and TME-modulating agents provide opportunities to translate these insights into biomarker-driven strategies for PTCLs, a group of lymphomas that still lack effective standard treatments.

### 2.1. Tumor-Intrinsic Immune Surveillance Escape Mechanisms

#### 2.1.1. Loss of Antigen Presentation

CD8+ T cell recognition of malignant cells requires the expression of tumor antigens on Major Histocompatibility Complex Class 1 (MHC-1) molecules [9]. PTCL tumor cells can evade immune surveillance by perturbing antigen presentation pathways, which reduce their immunogenic stimulus. High-throughput sequencing studies in peripheral T cell lymphoma, not otherwise specified (PTCL-NOS), have identified frequent loss-of-function mutations in pathways involved in immune surveillance, which include the components of MHC-1 (*HLA-A* and *HLA-B*) and the MHC Class II trans-activator *CIITA* [10,11]. Similar alterations have also been reported in extranodal NK/T cell lymphomas (ENKTL), where mutations in *HLA-A*, *β2M*, and *TAP1* correlate with more advanced disease stage [12], while approximately 50% of cases of adult T cell leukemia/lymphoma (ATLL) also harbor HLA Class 1 and/or *β2M* mutations [13,14].

Defects in the TAP1 gene, a component of the antigen processing machinery, and the HLA Class I genes (HLA-A, HLA-B, and HLA-C) enable tumor cells to evade immune destruction by CD8+ T cells due to impaired presentation of tumor antigens. Loss of MHC-II through the CIITA gene impairs the presentation of tumor-derived antigens to CD4+ T-helper cells, affecting dendritic cell priming and potentially impacting DC function cross-presentation. Together, these findings highlight antigen presentation defects as a recurring mechanism of impaired immune recognition in PTCL, enabling malignant clones to escape CD8+ T cell-mediated cytotoxicity, promoting disease progression.

#### 2.1.2. Downregulation of Adhesion Molecules

Although the downregulation of MHC class I molecules allows cancer cells to evade cytotoxic T cell-mediated killing, it may paradoxically render them more vulnerable to NK cell-mediated cytotoxicity, since the loss of MHC-I abrogates inhibitory signaling through Killer Immunoglobulin-like Receptors (KIRs) [15]. Accordingly, tumor cells have also evolved other mechanisms, such as the downregulation of adhesion molecules (CD58 and LFA-1) to escape killing by cytotoxic immune cells. Following engagement of the T cell receptor to MHC molecules, CD58 (LFA-3) and LFA-1 play key roles in the formation of immunological synapses, stabilizing and enhancing cell-to-cell adhesion [16,17]. Loss of CD58 in tumor cells has been shown to compromise CAR-T cell activity, driving tumor-intrinsic resistance to T cell killing [16]. Across T/NK cell lymphoma subtypes, CD58 expression is frequently lost, affecting >50% of cases in most PTCL categories, with the highest prevalence in ENKTL (83.3%), followed by PTCL-NOS (63.6%) and anaplastic large cell lymphoma (ALCL) (58%), underscoring its potential role in immune escape [10,18]. Similarly, genomic alteration of *CD58* has also been reported in ATLL, specifically in the acute form [13,19].

Beyond T cell interactions, CD58 is indispensable for NK cell-mediated cytotoxicity, and loss of CD58 in melanoma and B-lymphoblastic leukemia lines confers resistance to NK cell attack [16]. Putting this in the context of T cell lymphoma, the loss of CD58 expression may cause therapeutic resistance to monoclonal antibody therapies (mogamulizumab), which heavily rely on NK cell antibody-directed cell cytotoxicity (ADCC), underscoring its potential role in immune escape [18].

In addition, reduced expression of LFA-1, an integrin required for stable immune synapse formation, has been observed in ATLL cell lines, further contributing to tumor immune evasion [20]. These findings highlight adhesion molecule dysregulation as a recurrent mechanism of resistance in PTCL and support its evaluation as a biomarker to guide personalized T/NK cell-directed immunotherapies, including CAR-T and CAR-NK strategies.

#### 2.1.3. Overexpression of “Don’t Eat Me” Signals

Overexpression of CD47 was first identified in acute myeloid leukemia (AML), where its upregulation is linked to adverse outcomes [21]. Subsequently, CD47 overexpression has been documented across various cancers, including both hematologic and solid tumors [22,23]. CD47 contributes to tumor immune escape by binding to signal regulatory protein alpha (SIRPα) on myeloid cells, delivering a “don’t eat me” signal that prevents tumor cell phagocytosis by macrophages [24]. Consistent with this, single-cell transcriptomic profiling of nodal T follicular helper lymphoma angioimmunoblastic type (nTFHL-AI) demonstrated significantly higher expression of CD47 in malignant T-follicular helper (Tfh) T cells and B cells from relapsed/refractory (*r/r*) samples compared with newly diagnosed samples [25]. Relapsed/refractory samples exhibit a reduction in phagocytosis by myeloid cells, suggesting that CD47 overexpression confers a survival advantage during immune selection [25]. CD47 overexpression occurs in other PTCL subtypes, including PTCL-NOS and ALCL, although expression levels vary across tumor samples [26,27]. CD47’s established role in tumor immune evasion may represent a targetable vulnerability in personalized therapeutic approaches and has prompted the development of an anti-CD47 monoclonal antibody, which blocks the CD47-SIRPα interaction to promote phagocytosis of tumor cells. Multiple preclinical studies have now provided a rationale for testing this agent in TCL [23,27,28,29,30]. Interestingly, phase I studies of anti-CD47 (TTI-621) in 12 PTCL and 29 CTCL patients achieved an overall response rate (ORR) of 18% (2/11) and 21% (6/29), respectively (NCT02663518) [31], with 4 of 5 Sezary Syndrome (SS) patients having a prominent decrease in malignant clones 8 days after infusion [30]. This agent has also been demonstrated to potentiate responses to anti-PD-L1 checkpoint inhibitors in preclinical studies [32], which may provide further rationale for combination treatment [23,27,28,29,30]. Despite significant enthusiasm for CD47 targeting pathways given the therapeutic rationale and early clinical data, further trials to date across the spectrum of non-PTCL hematologic malignancies have not wholly delivered on this promise due to perceived lack of efficacy and/or potential toxicities, including significant anemia [33]. Future pathways for development in a competitive environment are not assured.

#### 2.1.4. Deregulated Apoptotic Pathways

In addition to extrinsic escape mechanisms, TCL cells utilize intrinsic survival strategies, most notably deregulated apoptotic pathways, which confer a selective survival advantage. Mutations of the *Fas* gene are frequently identified in ENKTL (50–60% of cases in two independent studies), resulting in resistance to Fas–FasL-mediated apoptosis and implicating this aberrancy in disease pathogenesis [34,35].

TNF-related apoptosis-inducing ligand (TRAIL)-mediated apoptosis represents another apoptotic pathway, initiated through binding of TRAIL to the death receptors DR4 and DR5. Notably, despite receptor expression on primary ATLL cells and cell lines, most are resistant to TRAIL-mediated apoptosis, indicative of a further means of apoptosis evasion [36]. TRAIL resistance in ATLL is mediated through the increased expression of anti-apoptotic proteins, including Bcl-xL, via activation of NF-κB by the Human T lymphotropic virus 1 (HTLV-1) Tax protein [36].

Further resistance results from overexpression of cellular FLICE inhibitory protein (c-FLIP), which also protects TCLs from apoptosis and contributes to tumor progression [37].

Deregulated apoptosis pathways may undermine the efficacy of immunotherapies, as T and NK cell-mediated cytotoxicity relies not only on perforin/granzyme but also on Fas-FasL and TRAIL-DR4/5 interactions to eliminate tumor cells [38]. Defects in these pathways may provide an additional layer of immune evasion and could potentially lead to resistance to T and NK cell-based therapies. Again, a more comprehensive understanding of these mechanisms could yield targeted therapeutic approaches that overcome this tumor survival advantage.

### 2.2. Tumor Microenvironment (TME)-Mediated Immunosuppression

The tumor microenvironment (TME) is a complex network of interactions between tumor cells and local cellular and non-cellular related factors. This includes non-malignant cells, such as immune cells, stromal cells, and fibroblasts, as well as surrounding blood vessels, the extracellular matrix (ECM), and other growth factors or cytokines [7].

#### 2.2.1. Cytokine and Chemokine Milieu of PTCLs

In PTCLs, the composition of the TME varies substantially between subtypes and is likely influenced by the lymphoma cell-of-origin (T-helper (Th)1, Th2, Th17, Tfh or Treg) [26,39,40]. The cell-of-origin defines the cytokine and chemokine profile, which governs immune cell recruitment and drives progressive remodeling of the TME. As an example, PTCL-NOS can be classified into two categories, namely PTCL-TBX21 and PTCL-GATA3 [40]. PTCL-TBX21 is characterized by a T-helper 1 (Th1)-dominant signature, with upregulation of Th1-related genes such as *IFNg*, *CXCL12*, *CCL2*, *CCL3*, *CCL6*, and *CCL11*. In contrast, GATA3-enriched cases exhibit overexpression of T-helper 2 (Th2) cytokines, including IL-4, IL-5, and IL-13 [40]. Additionally, the recently updated WHO designation of nodal T follicular helper lymphomas (nTFHLs) as a diagnostic entity, incorporating the previous diagnoses of angioimmunoblastic T cell lymphoma, follicular T cell lymphoma, and PTCL with T follicular helper phenotype [41], underscores the common feature within this group of a clonal proliferation of T-follicular helper (Tfh) cells, marked by overexpression of CXCL13 [42], IL-6, and IL-21 [43,44]. Furthermore, adult T cell leukemia/lymphoma (ATLL) is known to express FOXP3, a marker of regulatory T cells (Tregs) [45]. Tregs are known to secrete IL-10, IL-35, and TGF-β, which dampen the immune responses and potentially contribute to systemic immunosuppression [46].

Understanding the difference in the cytokine milieu may help elucidate the differences in TME signatures across different PTCL subtypes. This is shown where PTCL-TBX21 is associated with an enrichment in pro-inflammatory cells, likely due to the upregulated Th1 genes, evidenced by the enrichment of histiocytes in 89% of cases, with the remaining 11% consisting of a mixed immune cell infiltrate of lymphocytes, eosinophils, plasma cells, and histiocytes. This contrasts with PTCL-GATA3, which is characterized by low or absent immune cell infiltration [47,48]. The resulting cytokine skew suppresses TH1-mediated anti-tumor immunity, promoting an immune-excluded microenvironment which provides a molecular explanation for the paucity of inflammatory infiltrate observed in PTCL-GATA3. This may provide insights into the significantly better prognosis of PTCL-TBX21 cases, highlighting the need to convert the immune-excluded TME of PTCL-GATA3 into a more inflammatory one.

In nTFHL, recurrent cooperating lesions in *TET2*, *DNMT3A*, *IDH2*, and *RHOA*, together with aberrant PI3K-pathway activation, establish and sustain the TFH transcriptional program [49]. In n-TFHL angioimmunoblastic-type (nTFHL-AI), recent studies have revealed that the malignant Tfh population accounts for only 7–32% of the total cells [50,51]. Although they constitute only a minority of cells within the TME, the overexpression of Tfh cytokines [37,38] promotes the recruitment and expansion of B cells, plasma cells, and follicular dendritic cells (FDCs) [43,44]. Inducible T cell costimulator ligand (ICOSL), which is abundantly expressed on follicular FDCs, activated monocytes, and myeloid dendritic cells (mDCs), engages ICOS on malignant Tfh cells to drive proliferation and survival. This interaction activates downstream signaling cascades, including the PI3K–AKT and NF-κB pathways, which enhance metabolic fitness, cytokine secretion, and resistance to apoptosis. Beyond supporting malignant clone expansion, ICOS–ICOSL signaling reinforces the aberrant Tfh transcriptional program and promotes reciprocal crosstalk with surrounding immune and stromal cells, sustaining a permissive tumor microenvironment [50]. These data underscore the importance of cytokine-mediated TME remodeling in establishing a pro-tumorigenic niche.

Similarly, in ENKTL, single-cell transcriptomics have revealed that the tumor cells foster an immunosuppressive TME through enhanced expression of chemokines CCL3, CCL4, and CCL5, recruiting immune cells such as macrophages via their corresponding receptors CCR1 and CCR5 [52]. Additionally, ENKTL tumor cells can express DPP4, which cleaves CXCL2, CXCL9, and CXCL10, chemokines that recruit T cells and NK cells into the tumor microenvironment, depleting the number of anti-tumor effector cells [52].

#### 2.2.2. Immunosuppressive Cell Populations

Through chemokine/cytokine signaling, TCLs can recruit and expand multiple immunosuppressive cell types into the TME. In ENKTL, macrophages recruited into the TME can be skewed toward an immunosuppressive phenotype (M2-like) and interact with tumor-infiltrating T cells through the expression of immune checkpoints, such as CD80/CD86-CTLA4, PD-L1/PD-L2-PD-1, and Galectin-9-TIM3 [52,53]. Beyond TAMs, nTFHL-AI samples also show expansion of non-malignant CD4^+^FoxP3^+^ regulatory T cells (Tregs) compared with healthy lymph node controls. Tregs are known to secrete IL-10, IL-35, and TGF-β, which dampen the immune response and may contribute to tumor immune evasion [43,46,50,51].

Improving our understanding of the dynamic interplay among immune cell subsets is important for devising therapeutic approaches that can recalibrate the tumor microenvironment, shifting it from a state of immune suppression to one that facilitates durable antitumor immunity.

#### 2.2.3. Angiogenesis and Vascular Remodeling

TCL cells also adapt the TME through vascular remodeling, particularly by upregulating pro-angiogenic factors such as vascular endothelial growth factor (VEGF). In nTFHL-AI, increased VEGF expression in both lymphoma cells and endothelial cells causes remodeling of lymphoid structure, which develops an extensive network of high endothelial venules (HEVs) and FDCs adjacent to malignant Tfh cells [43,54,55]. In another PTCL subtype—anaplastic lymphoma kinase (ALK)-positive anaplastic large cell lymphoma (ALCL)—rearrangement of the ALK gene has also been shown to dysregulate the expression of Hypoxia-Inducible Factor (HIF-α) and subsequently VEGFA. In tumor xenografts, ALK knockdown was shown to reduce VEGFA expression, decrease tumor vessels in tumor xenografts, and control tumor growth [56]. Again, this pathogenic interaction highlights the therapeutic potential of disrupting the pro-tumorigenic microenvironment. Notably, this concept is supported by a case in which an nTFHL-AI patient achieved complete remission after treatment with the anti-VEGF antibody bevacizumab [57].

#### 2.2.4. Immune Checkpoint Pathways

The TME of TCLs is often characterized by profound T cell dysfunction and immune exhaustion. For example, in nTFHL-AI, recurrent loss of function in TET2 is found in >60% of cases, often preceding co-occurring RHOA G17V and IDH2 R172 mutations [50]. In mouse models, TET2 deficiency and RHOA mutations promote TFH polarization via ICOS/PI3K-MAPK signaling, leading to lymphoma development [58], while IDH2 further augments this process through TET inhibition [59]. These events promote chronic antigenic stimulation combined with progressive immune exhaustion, evidenced where CD8^+^ T cells frequently display heightened expression of immune checkpoint receptors, including PD-1, TIGIT, and CTLA-4. In the relapsed/refractory setting, the absolute number of CD8^+^ T cells declines relative to treatment-naïve disease; however, the residual population demonstrates further enrichment of these inhibitory receptors, suggesting progressive functional exhaustion in advanced disease [25,51]. In ENKTL, malignant cells also display marked upregulation of immune checkpoint molecules. Functional studies demonstrate that co-culture with normal T cells drives an exhaustion phenotype, characterized by increased checkpoint receptor expression and impaired IFN-γ production [52,53]. Similarly, ATLL tumor cells often upregulate immune checkpoint molecules, including PD-1, PD-L1, and CTLA-4, inhibiting T cell function and promoting immune evasion [13]. Notably, in ATLL, PD-L1 expression on tumor cells correlates with inferior outcomes [60,61], whereas PD-L1 expression on non-malignant microenvironmental cells is associated with significantly improved overall survival [60]. This dichotomy underpins the complexity of the tumor–immune interface: tumor-derived PD-L1 promotes immune evasion, while PD-L1 expression in microenvironmental cells may instead signify active anti-tumor immunity. These observations provide important context for the potential application of immune checkpoint inhibitors in ATLL, where prognosis remains poor.

In summary, accumulated evidence demonstrates that TCLs employ a diverse array of immune evasion strategies, including impaired antigen presentation, disruption of adhesion molecules, defective apoptotic signaling, and recruitment of immunosuppressive myeloid populations. These mechanisms not only shape the tumor microenvironment but also create subtype-specific vulnerabilities that may ultimately prove exploitable in a personalized manner. By integrating molecular and immunologic profiling, it is increasingly possible to stratify patients according to the dominant pathways of immune escape in their disease. This framework provides the biological rationale for the therapeutic strategies discussed in the following sections, including checkpoint inhibition, engineered cellular therapies, bispecific antibodies, and approaches that reprogram the tumor–immune interface.

## 3. Immunotherapy and Immune-Modulating Agents in TCL

### 3.1. Checkpoint Inhibitors in T Cell Lymphoma

Countervailing cancer immune evasion via blockade of immune checkpoint axes has revolutionized the management of many malignancies, including lymphomas, particularly Hodgkin lymphoma (HL). However, the use of these immune checkpoint inhibitors (ICIs), which leverage and augment T cell cytotoxic responses as a core mechanism of action, present a particular paradox when deployed against malignant T cells. In T cell lymphoma, PD-1 is not just an immune evasion pathway but also a cell-intrinsic constraint on malignant T cell proliferation, acting as a tumor suppressor in cell-based and murine models [62]. Mechanistically, PD-1 engagement restrains aberrant TCR-driven PI3K–AKT signaling and downstream MYC activation. Accordingly, PD-1 axis inhibition risks potentiating T cell lymphoma progression, a phenomenon termed the PD-1 paradox [62]. The PD-1 paradox was manifested in early trials of PD-1 inhibitors in ATLL, where multiple patients developed disease hyperprogression upon treatment, prompting study termination [63,64]. However, subsequent trials have not reproduced this disastrous complication (including in patients with ATLL) [65], and ICIs have demonstrable activity in some PTCL and CTCL patients, either as monotherapy or as part of combination regimens. These observations highlight the duality of PD-1’s role in T cell lymphomas and emphasize the importance of molecular and microenvironmental stratification to safely utilize checkpoint blockade in PTCL. Occasional “elite responders” achieve spectacular and durable responses. Consistent with the heterogeneity of TCL pathobiology, response rates vary considerably between disease contexts and studies, underscoring the urgent need to incorporate biomarker response predictors in patient selection.

#### 3.1.1. PD-1/PD-L1 Blockade in PTCL

ICI efficacy in PTCL varies significantly with disease subtype and context. Initial phase I and II studies of PD-1 inhibitor (pembrolizumab or nivolumab) monotherapy in relapsed/refractory PTCL reported widely varying ORRs, ranging from 17.6% in the AVAIL-T study to 40% in the study published by Lesokhin et al., reflecting participant heterogeneity and small cohort size (Table 1) [66,67,68]. Responses were only transient, and median progression-free survival (PFS) was <4 months for the patients with PTCLs in these trials.

The largest prospective trial of an ICI in PTCLs was reported by Shi et al. In it, the PD-1 inhibitor geptanolimab was deployed through a single-arm, phase II, multicenter study on relapsed/refractory PTCLs; of the 89 patients with reported data, the ORR was 40.4%, with a complete response rate (CRR) of 14.4% [69]. However, response rates varied significantly between PTCL subtypes, with the best response documented in patients with ENKTL (63.2%, 12/19), compared to ALK+ALCL (53.8%, 7/13), ALK-negative ALCL (42.9%, 3/7), and PTCL-NOS (17.9%, 5/28) [69]. Additionally, several non-randomized studies have investigated the use of ICIs as maintenance therapy following autologous stem cell transplantation (auSCT), demonstrating the safety of this strategy, but with evidence of long-term efficacy still awaited [70,71]. See Table 1 for additional details on clinical evidence for ICIs in PTCLs.

Of importance, patients with relapsed/refractory ENKTL defy the broader PTCL trend, consistently demonstrating high rates of response to PD-1 axis ICIs across multiple studies [69,72,73,74,75,76,77,78] (Table 1). ORRs in these studies frequently exceed 50%, including CR rates of up to 31.3%. However, response duration is highly variable, with median PFS not reached in several studies, whereas others reported modest PFS rates averaging several months. In ENKTL, superior response to ICI is thought to be mediated by the near-universal association of the disease with Epstein–Barr Virus (EBV). EBV plays a fundamental role in the pathogenesis of ENKTL, and EBV-encoded proteins expressed during viral latency can cause aberrant expression of NF-κB, PI3K/AKT, JAK/STAT, and other signaling pathways [79]. Perturbed JAK/STAT signaling can upregulate PD-L1 expression on tumor cells generating an immunosuppressive tumor microenvironment [80]. Additional evidence suggests that EBV-derived miRNAs can also promote PD-L1 expression [81]. These phenomena render these tumors dependent on immune evasion and thus more vulnerable to checkpoint inhibition.

Furthermore, ICI therapy may augment the presentation viral antigens, enhancing immune stimulation and anti-tumor immune responses [82]. These findings prompt consideration of whether EBV status is a potential biomarker of ICI response in PTCLs; however, this conjecture remains incompletely addressed by existing data. Given the challenge of recruiting sizable cohorts of PTCL patients, studies have not generally reported responses stratified by EBV status. In non-Hodgkin lymphoma (NHL) more broadly, Kim et al. reported that EBV positivity was associated with improved ICI responses, though the responder cohort was enriched with ENKTL patients (*n* = 6/7 responders), limiting generalizability [75].

Beyond EBV positivity, other biomarkers of ICI response have been explored, but a robust predictive method remains elusive.

#### 3.1.2. Immune Checkpoint Expression

*PD1*, *PD-L1*, and *PD-L2* expression has been repeatedly investigated, as have levels of soluble PD-L1. However, assessment methods and scoring techniques vary considerably, as does the utility of these metrics across different lymphoma subtypes. Unfortunately, no cogent tool based on PD-L1/PD-L2 tumor or TME expression has proven of sufficient clinical benefit, and documented responses to PD-L1-negative tumors indicate the lack of discriminative power for these pathological factors [83]. Some studies do indeed report superior outcomes in cases with higher PD-L1 expression; however, attribution is difficult as responders are enriched for ENKTL cohorts [69]. Overall, as a biomarker of ICI response, PD1/PD-L1/PD-L2 expression assessment suffers from a lack of standardization in assessment, biological complexity, variability across disease subtypes, and inconsistent clinical correlation, and it cannot be considered a reliable indicator of ICI response in PTCLs.

#### 3.1.3. Tumor Mutational Burden

Tumor mutational burden (TMB), defined as the total number of somatic mutations per coding unit of the genome (typically as mutations per megabase (mut/Mb)), has emerged as a biomarker of ICI response in multiple solid organ cancer contexts [84,85,86]. This is thought to reflect a proportional relationship between TMB and the degree of tumor neoantigen expression and, therefore, immunogenicity [87]. However, TMB has yielded inconsistent results in hematological malignancies, where the mutational burden is highly variable between disease entities (but trends lower than that seen in archetypical ICI responsive solid cancers like melanoma) [88,89,90,91]. Although some studies have demonstrated a potential relationship between TMB and ICI response in some lymphoma subtypes, data are neither robust nor consistent [92]. Unsurprisingly, such data are far less developed in the PTCL context specifically, but panel-based sequencing (with its attendant limitations) suggests a lower TMB in TCLs compared to B cell lymphomas [91].

#### 3.1.4. Microsatellite Instability (MSI) and Deficient Mismatch Repair (dMMR)

Microsatellites are repetitive genomic motifs of 1–6 nucleotides distributed across the genome and contribute to chromatin organization, DNA stability, and gene expression regulation. However, their repetitive nature predisposes microsatellites to replication errors during DNA synthesis. Ordinarily, such errors are corrected by the DNA mismatch repair (MMR) system. When this repair system is dysfunctional—termed deficient mismatch repair (dMMR)—microsatellite sequences accumulate insertion–deletion mutations, resulting in microsatellite instability (MSI) [93]. MSI-high and dMMR phenotypes are associated with greater mutational loads, leading to an elevated neoantigen burden, which can enhance tumor immunogenicity and drive strong responses to immune checkpoint inhibition in some cancers [94,95]. However, dMMR and MSI rarely occur in lymphoma, and in contrast to solid organ cancers, these factors cannot be used to predict response to ICIs for hematological cancer [96,97,98]. Again, the paucity of any data in PTCLs precludes meaningful discussion, though MSI has been shown in a subset of monomorphic epitheliotropic intestinal T cell lymphoma (MEITL) and ATLL cases and could be explored further [99,100].

#### 3.1.5. Chr 9p Structural Variants

Chromosome 9p24.1 includes the loci of *CD274* (encoding the PD-L1 checkpoint) and *PDCD1LG2* (encoding PD-L2), and structural variants (SVs) affecting this region have proven amongst the most reproducible indicators of ICI response in lymphoma. In classical Hodgkin lymphoma, copy number gains, focal amplifications, and translocations at 9p24.1 are strongly associated with overexpression of PD-L1/PD-L2 and can be coupled with JAK2-driven STAT signaling, further enhancing PD-L1 transcription. These variants induce PD-1 axis dependency and underpin the efficacy of PD-1 inhibitors in this disease, with objective response rates exceeding 65–85% across multiple trials [101,102,103,104].

In non-Hodgkin lymphomas, 9p24.1 SVs are less common but remain clinically relevant. In primary mediastinal B cell lymphoma (PMBCL), 9p24.1 structural changes are also associated with high PD-L1 expression and impressive ICI responses [105,106]. In PTCLs, the frequency of 9p24.1 alterations is incompletely defined, but published genomic datasets suggest they occur at lower prevalence than in Hodgkin lymphoma or PMBCL [107]. Rates vary significantly between TCL subtypes, however, with high rates in EBV-driven disease generally reflected in the ENKTL cohort, where 9p24.1 SVs were found in 23% of cases, as well as in 15% of cases of EBV-positive PTCL-NOS [107]. Data can be inconsistent, however, as Ng and colleagues showed only low rates of 9p24.1 SV in EBV-driven T/NK cell lymphomas despite documented overexpression of PD-L1, suggesting alternative mechanisms leading to checkpoint upregulation [108]. SVs involving *CD274* are also documented in mycosis fungoides with large cell transformation, with small numbers of patients demonstrating high rates of disease response, though these responses appeared to be ephemeral [109]. In ATLL, a variety 9p24.1 SVs have been documented in approximately 10–25% of patients, but correlation with ICI response is unknown [110,111]. Though a promising biomarker of ICI response in HL, ENKTL, and PMBCL, the independent predictive power of chromosome 9p SVs for ICI response in TCL remains unproven, though some data suggest this possibility.

#### 3.1.6. PD-1/PD-L1 Blockade Combination Regimens in PTCL

Numerous efforts have been made to augment responses to ICIs in TCL by deploying them in combination with other targeted therapeutics or with conventional cytotoxic therapies (Table 1).

The addition of the histone deacetylase inhibitor (HDACi) romidepsin to pembrolizumab has been investigated in relapsed/refractory PTCL, with the most recent data reporting high response rates, particularly in the nTFHL subtypes (ORR 85.7%, CRR 57.1%) [112]. With a median duration of follow-up of 40 months, median PFS for nTFHL patients was not reached, but it was only 1.23 months in the PTCL NOS cohort [112]. Other pembrolizumab combination trials include partnering with pralatrexate (NCT03598998) and the multi-arm Embolden study with pembrolizumab added to decitabine, decitabine and pralatrexate, or pralatrexate (NCT03240211). Embolden reported initial data in 2022, demonstrating responses in heavily pre-treated patients (2/3 patients receiving the triplet regimen responded), but further data are awaited to draw meaningful conclusions on safety and efficacy [113]. In relapsed/refractory ENKTL, a further multi-agent regimen (PCET) is under investigation, utilizing the HDACi chidamide, etoposide, and thalidomide combined with the PD-1 target monoclonal antibody toripalimab. Early data are extremely limited (*n* = 3), but all patients responded (2 CRs, 1 PR). [114]

Additional combinations under active study in TCL include pembrolizumab with brentuximab vedotin (NCT05313243); camrelizumab (PD-1 inhibitor) with pegasparaginase and apatinib (VEGFR2 inhibitor) in ENKTL (NCT04366128); nivolumab with chemotherapy for relapsed ALCL (NCT07013565); duvelisib and nivolumab in mycosis fungoides (MF) and Sezary Syndrome (SS) (NCT04652960); sintilimab (PD-L1 inhibitor) with gemcitabine and oxaliplatin (NCT04127227); and durvalumab with different combinations of pralatrexate, romidepsin, and oral azacitidine (NCT03161223).

**Table 1:** Studies evaluating checkpoint inhibitor therapy in peripheral T cell lymphoma, organized by monotherapy versus combination regimens and by disease subtype.

Trial and Phase	Line of Therapy	No of Patients (TCL Cohort)	Regimen	ORR	CR	Median PFS	OS	Notes	Reference
**Checkpoint inhibitor monotherapy studies—mixed PTCL**									
**AVAIL-T** **NCT03046953**	Median 3 prior lines Range 1–7	Total: 34 AITL: 11 PTCL NOS: 17 ENKTL: 4 ALCL: 1 Transformed MF: 1	Avelumab monotherapy	17.6%	0%	2.9 months	8.9 months	N/A	[65]
**Phase I** **NCT01592370**		5	Nivolumab monotherapy	40%	0%	14 weeks		N/A	[67]
**Phase II** **NCT03075553**	Median 2 prior line Range 1–6	12 AITL: 6 PTCL NOS: 3 ALCL ALK-: 1 EATL: 1 HSTCL: 1	Nivolumab monotherapy	33%	16.7%	2.7 months	6.7 months	Median DOR: 3.6 months Hyperprogression in 4 patients (3 AITL and 1 HSTCL)	[114]
**GXPLORE-002** **Phase II** **NCT03502629**	Median 2 prior line	102 (89 in full analysis set) PTCL NOS: 41 ENKTL: 23 ALCL ALK-: 12 ALCL ALK+: 7 EATL: 3 MF: 3 Others: 12	Geptanolimab monotherapy	40.4%	14.6%	2.7 months	14.6 months	DOR: 11.4 months ORR by disease subtype ENKTL: 63.2% ALCL ALK-: 53.8% ALCL ALK+: 42.9% PTCL NOS: 17.9% PD-L1 ≥50% enriched (ORR 53.3% vs. 25%)	[68]
**Phase II** **NCT02535247**	Median 2 prior lines Range 1–9	18 (13 evaluable) PTCL NOS: 7 TFH: 4 Transformed MF: 3	Pembrolizumab monotherapy	33%	27%	3.2 months	10.6 months	Halted early after futility analysis	[66]
**Pembrolizumab maintenance post ASCT** **Phase II** **NCT02362997**	N/A	21 PTCL NOS: 11 AITL: 4 ENKTL: 3 ALCL ALK-: 2 MEITL: 1	Pembrolizumab monotherapy Post ASCT maintenance study	N/A	N/A	18-month PFS: 83.6%	18-month OS: 94.4%	N/A	[70]
**Duvelisib maintenance post ASCT** **Phase II** **NCT04331119**	N/A	12 (5 PTCL) ALCL: 3 PTCL NOS: 1 AITL: 3	Duvelisib monotherapy Post ASCT maintenance study	N/A	N/A	Note reported	Not reported	N/A	[69]
**Checkpoint inhibitor monotherapy studies—ENKTL**									
**ORIENT-4** **Phase II** **NCT03228836**	Median 3 prior lines	28	Sintilimab monotherapy	75%	21.4%	Not reported	NR 2yr OS: 78.6%	Median DOR: 4.1 months Pseudoprogression in 17.9%	[75]
**Avelumab** **Phase II** **NCT 03439501**	Median ≥2 prior lines	21	Avelumab monotherapy	38%	24%	2.7 months	NR	Response by PDL1 expression: High PD-L1: 73% Low PD-L1: 0%	[77]
**GEMSTONE-201** **Phase II** **NCT03595657**	Median 1 prior lines	80	Sugemalimab monotherapy	44.9%	35.9%	Not reported	NR 18-month OS: 57.9%	Median DOR: NR	[115]
**Pembrolizumab** **(Retrospective case series)**	Median 2 prior lines Range 1–5	7	Pembrolizumab monotherapy	100%	71.4%	Not reported	Not reported	Non-trial study	[71]
**Pembrolizumab** **(Retrospective case series)**	Median 4 prior lines Range 3–10	7	Pembrolizumab monotherapy	57%	28.6%	4.8 months	5 months	Non-trial study DOR: 4.1 months	[74]
**Pembrolizumab** **(Retrospective case series)**		14	Pembrolizumab monotherapy	44%	35.7%	Not reported??	N/A	N/A	[74]
***Checkpoint inhibitor combination studies—Mixed PTCL***									
**Pembrolizumab + Romidepsin** **Phase I/II** **NCT03278782**	Median 2.5 prior lines	20 (14 evaluable for efficacy) PTCL NOS: 7 TFH: 15 AITL: 2 Transformed MF: 3 ALCL: 3 ENKTL: 2	Pembrolizumab + Romidepsin	50%	35.7%	Not reported	Not reported	Hyperprogression occurred in 2 patients	[116]
**Camrelizumab + Apatinib** **Phase II** **NCT03701022**	Median 3 prior lines Range 1–6	ENKTL: 7 PTCL NOS: 6 AITL: 5 ALCL ALK-: 2	Camrelizumab + Apatinib	30%	10%	5.6 months	16.7 months	DOR: not reached Patients with PD-L1 expression ≥ 50% vs. <50% ORR (66.7% vs. 40%). The two patients with the highest PD-L1 expression showed PFS of 18.2 and 22.3 months, respectively.	[117]
**PCET** **(Retrospective case series)**	Median 2 prior lines Range 1–6	ENKTL: 3	PCET: toripalimab + chidamide + etoposide + thalidomide	100%	66.7%	Not reported	Not reported	Non-trial study	[113]

Abbreviations: AITL, angioimmunoblastic T cell lymphoma; ALCL, anaplastic large cell lymphoma; ALK, anaplastic lymphoma kinase; ALK-, ALK-negative; ALK+, ALK-positive; ASCT, autologous stem cell transplantation; CR, complete response; DOR, duration of response; EATL, enteropathy-associated T cell lymphoma; ENKTL, extranodal natural killer/T cell lymphoma; HSTCL, hepatosplenic gamma delta T cell lymphoma; MEITL, monomorphic epitheliotropic intestinal T cell lymphoma; MF, mycosis fungoides; N/A, not applicable; NR, not reached; ORR, overall response rate; OS, overall survival; PCET, PD-1 antibody + chidamide + etoposide + thalidomide; PD, progressive disease; PD-L1, programmed death-ligand 1; PFS, progression-free survival; PTCL, peripheral T cell lymphoma; PTCL NOS, peripheral T cell lymphoma not otherwise specified; TCL, T cell lymphoma; TFH, T-follicular helper.

Despite a compelling biological rationale, clinical responses to PD-1 and PD-L1 blockade in PTCLs have been inconsistent overall, and most promising results have been largely confined to ENKTL and TFH-derived subtypes. Most PTCL-NOS and ALK-negative ALCLs derive little benefit, and PD-1-associated hyperprogression has been observed in ATLL and occasionally in other subtypes. Even within CTCL, response rates are variable, reflecting the influence of tumor-intrinsic genotype, cytokine milieu, and immune context [115]. Current predictive biomarkers have shown limited reliability, underscoring that checkpoint efficacy in PTCL cannot yet be confidently forecast. Collectively, these findings indicate that checkpoint inhibition alone is insufficient for most PTCL and highlight the need for deeper biomarker discovery and rational combination strategies. Emergent work examining the interplay of molecular profiling and PD-L1 expression, for example, suggests a potential way to focus research efforts [116]. Integration of immune-gene profiling, spatial transcriptomics, and multi-omic signatures will be critical to define the subsets most likely to benefit and to convert these biologic insights into effective, personalized immunotherapeutic regimens.

### 3.2. Engineered Cellular Therapies (CAR-T, CAR-NK)

**Figure 2:**
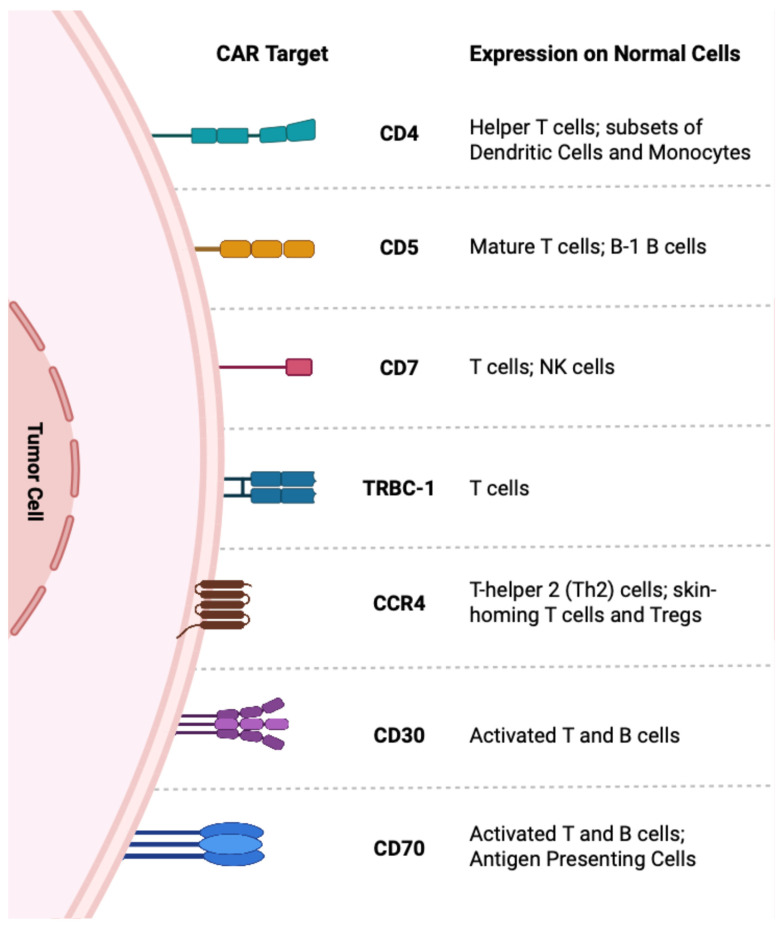
Surface antigens currently under clinical or preclinical investigation as CAR-T targets in peripheral T cell lymphomas. Pan-T cell antigens (CD4, CD5, CD7) present challenges due to T cell aplasia and fratricide, prompting safety-switch strategies and gene-editing approaches. More selective targets (TRBC1/2) exploit clonal restriction of the malignant TCR to spare healthy T cell compartments. Additional targets with more restricted or disease-specific expression, such as CD30, CCR4, and CD70, may improve therapeutic windows. These approaches highlight both the opportunities and unique challenges of CAR-T therapy in T cell lymphoma.

Over the past decade, adoptive cell transfer (ACT) therapy has demonstrated significant efficacy in treating various cancers, particularly in B cell malignancies. Anti-tumor lymphocytes used in ACT therapy include naturally occurring tumor-infiltrating lymphocytes (TILs) or lymphocytes that have been genetically engineered to express an anti-tumor T cell receptor (TCR) or chimeric antigen receptor (CAR). CAR-T cell therapy has emerged as the leading advance, distinguished by its unprecedented efficacy in relapsed/refractory B cell malignancies [117,118,119]. Recognition of tumor antigens by CARs is achieved through an antigen-binding domain commonly derived from the variable regions of antibodies [120]. Therefore, antigen recognition by CAR bypasses the need for peptide presentation on MHC molecules, where its downregulation in PTCL samples is one of the mechanisms by which tumors evade host immunity, as described above. Given this, there is considerable interest in adopting CAR-T cell therapy for the treatment of T cell lymphomas. However, there are several challenges due to the sharing of antigens between malignant cells and normal T cells, which can lead to the depletion of healthy T cells, causing profound immunosuppression (T cell aplasia), CAR-T cell fratricide (self-killing of CAR-T cells), and contamination of CAR T cell products during manufacturing. In the following section, we will review CAR-T and CAR-NK targets (Figure 2) currently under clinical investigation for PTCLs (Table 2) and summarize their therapeutic potential. To date, no CAR-/TCR-T therapies have been approved for this disease group.

#### 3.2.1. CD4

CD4 is a membrane glycoprotein expressed on T cells that serves as a co-receptor for the T cell receptor (TCR). In healthy individuals, approximately 40–50% of T lymphocytes are CD4+ T cells [121,122]. Upon antigenic stimulation, these cells differentiate into diverse T-helper subsets that coordinate and regulate adaptive immune responses [122]. In PTCL, the malignant clone is most often CD4^+^, representing approximately two-thirds of cases, whereas CD8^+^ PTCLs account for only 15–25%, with the remainder typically double-negative and, more rarely, double-positive [123]. Owing to this, Pinz et al. developed CAR-T cells targeting CD4 and demonstrated significant anti-tumor efficacy both in vitro against the Karpas 299 ALCL line and in mouse xenograft models [124]. Despite the remarkable efficacy in preclinical studies, concerns arise surrounding the safety of depleting healthy CD4+ T cells in patients, as CD4+ T cells play a crucial role in providing help to multiple immune effectors, including antibody production by B cells, licensing of dendritic cells to enhance CD8+ T cell functions, and other direct effector functions through cytokine secretion. Unlike the depletion of B cells, which can be tolerated or compensated through intravenous immunoglobulin infusion, the depletion of CD4+ T cells leads to profound immunosuppression, rendering patients vulnerable to opportunistic infections, a situation akin to HIV-associated depletion of CD4+ T cells. To circumvent this, the group has engineered a CD52 alemtuzumab safety switch to deplete the infused CD4 CAR-T cells and reverse the CD4+ T cell aplasia, shortening the window of immunosuppression [125]. Currently, there are multiple ongoing phase I trials of CD4 CAR-T cells in T cell lymphoma (NCT04162340, NCT04712864, NCT03829540). Preliminary data from the first three patients in the NCT04162340 clinical trial showed that two patients treated with CD4 CAR-T cells engineered to secrete an IL-15/IL-15Rα complex achieved durable complete remissions for 15 months and 8 months, respectively, and the third patient achieved a durable partial response (PR) [126]. Importantly, none of the three patients experienced severe opportunistic infections, neurotoxicity, or grade 3 and above cytokine release syndrome (CRS), indicating that the CD4 CAR-T cells were well tolerated in these patients. Better insights into the safety and tolerability of CD4 CAR-T cells are anticipated with the enrolment of more patients and the publication of these phase I studies.

#### 3.2.2. CD5

In 2015, CD5-targeting CAR-T cells were developed by Mamonkin et al. to target T cell acute lymphoblastic leukemia (T-ALL) and T cell lymphomas [127]. CD5 is a surface glycoprotein expressed on all mature T cells and a subset of B cells, serving as a negative regulator of cellular activation [128]. Reflecting their post-thymic origin, malignant clones in PTCL frequently express CD5, with studies reporting positivity in ~63% of cases, though there is notable variation in expression patterns between subtypes [129]. Nodal Tfh lymphoma, PTCL-NOS, and ATLL have the highest proportion of patients with CD5-expressing tumors (>75%), whereas low expression is observed in ENKTL, breast-implant-associated (BIA) ALCL, and hepatosplenic TCL (HSTCL) [129]. Despite CAR-T cell fratricide caused by CD5 expression on mature T cells, Mamonkin et al. successfully generated CD5 CAR-T cells, as the surviving CAR-T cells downregulated surface CD5 expression [127]. Importantly, this loss of CD5 did not compromise the cells’ effector function, which demonstrated preserved cytotoxicity in vitro and in vivo against preclinical models of T-ALL and T cell lymphoma. Supporting this, CD5-knockout CAR-T cells have been shown to exhibit augmented CAR-T cell function, consistent with CD5’s role as a negative regulator of T cell activation [130].

While CAR-T fratricide can be mitigated by gene editing or expanding CD5-downregulated T cells, off-tumor targeting of normal T cells remains a concern. The risk of prolonged T cell aplasia has also prompted the development of safety switches in CD5 CAR-T cells. Inclusion of CD52 or truncated Epidermal Growth Factor Receptor (tEGFR) safety switches allows for antibody-mediated depletion (anti-CD52 and anti-EGFR, respectively) of these CAR-T cells in the event of unacceptable toxicity [131,132].

A recently published phase I clinical trial of autologous CD5 CAR-T cells (NCT03081910) targeting relapsed/refractory T cell lymphomas has shown a tolerable safety profile, with no patient experiencing CRS of grade 3 or higher [133]. However, three out of nine patients experienced prolonged cytopenias that persisted beyond 28 days. Although prior case reports described remarkable responses of CD5 CAR-T cells in ALK^+^ ALCL [134] and T cell lymphoblastic lymphoma (T-LBL) [135], outcomes in this clinical trial were modest. Among nine treated patients, four had progressive disease (PD), one achieved stable disease (SD) but subsequently died following salvage therapy, one achieved a partial response (PR), and three achieved complete responses (CRs). At the end of follow-up, only two patients remained alive: one with nTFHL-AI who attained CR and subsequently underwent allogeneic hematopoietic stem cell transplantation (allo-HSCT) and another who relapsed after an initial CR. Six patients died of lymphoma progression and one from allo-HSCT complications. This limited efficacy may reflect the heavily pre-treated nature of the cohort (median of five prior therapies, up to eighteen in one patient), which likely compromised T cell fitness for autologous CAR-T manufacturing. Notably, clinical responses were confined to patients treated with CAR-T products generated using a shortened manufacturing protocol. This suggests that a minimally differentiated CAR-T product may be crucial for expansion and persistence. Additional challenges with autologous CD5 CAR-T manufacturing for T cell lymphoma include malignant cell contamination, as observed in a study from Hill et al., in which a CAR-T product had to be abandoned before infusion, as well as the potential for a long interval from apheresis to CAR-T cell infusion, with two patients dying from disease progression in this study while awaiting receipt of CAR-T cells [133].

Allogeneic CD5 CAR-T cells offer potential benefits by reducing the time to CAR-T infusion, ensuring T cell fitness, and preventing malignant clone contamination. Encouragingly, a phase II trial of allogeneic CD5 CAR-T cells in *r/r* T-ALL (NCT05032599) demonstrated a 100% response rate in 16 patients, suggestive also of their potential utility in the treatment of CD5-positive PTCL [136].

#### 3.2.3. CD7

CD7 is a pan-T cell antigen expressed by most peripheral T cells [137], NK cells, and in more than 95% of T-lymphoblastic leukemia/lymphoma (T-ALL and T-LBL) [138]. Its expression in PTCL tumors varies considerably between subtypes, with 25% to 79% of cases being positive or partly positive [139]. In contrast to CD5-targeted CAR-T cells, which undergo only transient fratricide followed by complete loss of CD5 expression on the CAR-T population, CD7-targeted CAR-T cells are vulnerable to substantial fratricide following CAR transduction due to the high physiologic expression of CD7 on normal T cells [140]. Consequently, several strategies have been developed to yield fratricide-resistant CD7 CAR-T cells, including knocking out CD7 via genome editing [140], intracellular protein expression blockade (PEBL) [138], and pharmacologic inhibition of CAR signaling [141]. These approaches enabled the successful generation of CD7-targeting CAR-T cells, which possess significant anti-tumor activity both in vitro and in vivo. Interestingly, Lu et al. were able to develop a “naturally selected” CD7-targeting CAR-T cell therapy without additional manipulation of the T cell genome or culture conditions [142]. These naturally selected CAR-T cells undergo self-destruction (fratricide), and those that survive this process become the final CAR-T product. This study demonstrated that despite lower folds of expansion in these naturally selected CD7-targeting CAR-T (NS7CAR) cells, manufacturing was still able to be scaled successfully for clinical application. The researchers hypothesized that the surviving NS7CAR T cells had escaped fratricide due to antigenic masking or intracellular sequestration of CD7 by the CAR. Promisingly, in this phase I study of NS7CAR-T cells in T-ALL and T-LBL patients (NCT04572308), 19 out of 20 patients achieved minimal residual disease-negative (MRD-negative) CR in the bone marrow [142].

Thereafter, a further trial evaluated NS7CAR T cell therapy in CD7-positive *r/r* PTCL (NCT04928105) [143]. Preliminary data from the first five patients treated showed a remarkable response, where three patients achieved CR and one had a PR. CD7 CAR-T cells also demonstrated a manageable safety profile in these patients, with no patient experiencing grade ≥ 3 CRS or neurotoxicity. However, prolonged depletion of healthy T and NK cells following infusion of CD7 CAR-T cells remains a concern. While not observed in the PTCL trial, a follow-up study in T-ALL reported infections in 6 of the 12 patients who did not undergo allogeneic SCT consolidation, including 4 infection-related deaths [144]. These findings underscore the need for larger patient cohorts and extended follow-up to fully elucidate the safety and efficacy of CD7 CAR-T cells in PTCL. An additional limitation is CD7 downregulation, a frequent phenomenon in PTCLs [145]; hence, patient stratification is critical to ensure that the benefits of CD7 CAR-T cell therapy outweigh the risks. Ongoing clinical trials (NCT05290155, NCT05059912, NCT06925464, NCT04004637, NCT05979792, NCT04480788, NCT04264078, NCT05377827) will provide valuable insights into the efficacy and safety of this treatment.

#### 3.2.4. TRBC-1

The T cell receptor (TCR) is a transmembrane complex consisting of two distinct protein chains. In humans, approximately 95% of T cells express an ⍺β TCR, formed by alpha (α) and beta (β) chains, and the remaining 5% of T cells express a ɣδ TCR, composed of a gamma (ɣ) and a delta (δ) chain. Within ⍺β T cells, the β chain is unique to each T cell clone and is structured into variable (V), diversity (D), joining (J), and constant (C) regions. The constant (C) region of the beta (β) chain is encoded by two mutually exclusive genes, TRBC1 and TRBC2. Hence, T cells expanding from the same clone express the same TCRβ constant gene, either TRBC1 or TRBC2. Because PTCL arises from mature T cells, clonal lymphoma cells typically exhibit monoclonality at the TRBC locus (when derived from ⍺β T cells), with homogeneous expression of either TRBC1 or TRBC2 [146,147,148]. Leveraging this clonality, Maciocia et al. developed TRBC1-targeting CAR-T cell therapy TRBC1+ tumors [146], aiming to ensure that healthy T cells expressing TRBC2 remained unaffected. Preliminary in vitro studies affirmed this hypothesis, and the investigators demonstrated TRBC1-targeting CAR-T cell therapy depleted only TRBC1-expressing tumors and normal T cells, sparing TRBC2-expressing T cells. Notably, in the phase I/II trial of TRBC1-CAR T cell therapy (NCT03590574) [149], the CAR-T therapy was well tolerated, with no severe adverse events, as evidenced by the absence of immune cell-associated neurotoxicity syndrome (ICANS) or dose-limiting toxicity. Only one patient experienced grade 3 CRS, which resolved within three days. Out of the nine evaluable patients, four achieved a complete response (CR) and two had a partial response (PR). Unfortunately, only two of the four that achieved a complete response had an ongoing remission at 15 and 18 months, whereas all other patients eventually developed progressive disease. Given the inability to readily detect the CAR-T cells in the peripheral blood, the lack of durable remission is likely attributable to limited expansion and persistence of the CAR-T cells. Concerningly, a recent study suggested that normal T cells may eliminate anti-TRBC1 CAR-T cells, potentially contributing to their limited persistence [150]. Additional contributors to poor CAR-T persistence may include fratricidal killing by non-malignant T cells, a bias toward effector-like differentiation, and the compromised fitness of autologous T cells rendered dysfunctional and exhausted by prior therapies [151]. Nevertheless, TRBC1-targeting CAR-T cell therapy holds promise as a novel treatment for PTCL patients, and sustained remission may be achieved by improving CAR-T cell manufacturing (e.g., using allogeneic CAR-T cells or generating Tstem-like CAR-T cells). Given TRBC1 targeting excludes a substantial number of patients to be eligible, the development of TRBC2-targeting CAR-T cell therapy is also currently underway for TRBC2-positive PTCL tumors [152]. Such approaches will spare a considerable fraction of the healthy residual T cell population, mitigating the risks of T cell compartmental ablation seen with pan T antigen targeting strategies.

#### 3.2.5. CD30

CD30 is a membrane glycoprotein from the tumor necrosis factor (TNF) receptor family and is frequently overexpressed in classical Hodgkin lymphoma (HL) and ALCL [153,154], with expression observed in 93–100% of cases in both ALK-positive and ALK-negative ALCL tumors [139,155]. Multiple studies have also characterized its expression in other PTCL subtypes, including PTCL-NOS (16–58%), nTFHL-AI (21.42–63%), ATLL (39–55.5%), enteropathy-associated T cell lymphoma (EATL, 50–100%), ENKTL (46–70%). In contrast, MEITL and HSTCL show no detectable CD30 expression on tumors [139,155,156]. Given its restricted expression of only a small subset of activated T and B cells in healthy adults [154], CD30 has been regarded as a safe and attractive tumor target compared to other pan-T cell markers, leading to the successful development of brentuximab vedotin, a CD30 antibody–drug conjugate, which is discussed in more detail later in this review [157,158,159,160]. However, outside of durable responses in patients with ALCL, responses to brentuximab can be brief in other PTCL subtypes, mirroring the 22% 5-year PFS reported in HL. On the other hand, CAR-T cells may provide more durable disease control due to their persistence and formation of memory subsets [161]. Consistent with this, a patient with EATL who received CD30 CAR-T cell therapy made from allogeneic-donor stem cells achieved a complete response and remained in remission for 24 months [162].

In a phase I dose escalation study of CD30 CAR-T cells by Ramos et al. (NCT01316146), one of two enrolled ALCL patients achieved CR, and no CAR-T infusion-related toxicities were observed [163]. By contrast, Wang et al. reported treatment-related fatalities [164], including a Hodgkin lymphoma patient with high tumor burden who died of pleural hemorrhage with massive CD30 CAR-T infiltration and an ALCL patient who died of infection, possibly linked to targeting of normal CD30^+^ T cells. Nevertheless, two of three ALCL patients in that trial achieved durable CRs lasting over one year [164].

CD30 CAR-T cells have also been evaluated as consolidation following autologous HSCT with BEAM conditioning in a mixed patient population with either Hodgkin lymphoma (HL) or PTCL [98] (NCT02663297). While this approach yielded encouraging outcomes in the HL cohort (2-year PFS 73%), five of six patients with PTCL (four with ALCL, one with nTFHL-AI, and one with PTCL-NOS) died during follow-up, four from disease progression and one from unrelated lung cancer, highlighting the limited efficacy for TCL in this setting. Seeking to improve this, Grover et al. (NCT03602157) are evaluating whether co-expressing CCR4 on CD30 CAR-T cells can enhance tumor homing [165]. Ultimately, larger PTCL-specific studies are required to define the therapeutic potential of CD30 CAR-T cells and to clarify the biological basis for the differing responses observed between HL and PTCL.

#### 3.2.6. CD70

CD70, the ligand for the costimulatory receptor CD27 within the Tumor Necrosis Factor (TNF) receptor–ligand superfamily, is normally expressed on antigen-presenting cells and activated T cells [166,167]. However, dysregulated expression can drive aberrant CD27-CD70 signaling, which has been implicated in various solid tumors and hematological cancers, including T cell lymphomas [168]. CD70 is aberrantly overexpressed across several T cell lymphoma subtypes, notably in cutaneous T cell lymphoma (CTCL), including MF and primary cutaneous ALCL, and in certain PTCL subtypes, such as PTCL-NOS, systemic ALK-negative ALCL, nTFHL-AI, and acute-type ATLL [169,170,171]. Functionally, CD70 has also been implicated in promoting tumor growth and facilitating immune escape [168]. CD70’s direct pathophysiological role coupled with its overexpression in T cell lymphomas make it a promising candidate for targeting.

Several CD70-directed therapeutics have been developed, including the antibody–drug conjugate SGN-CD70a [171], the monoclonal antibody cusatuzumab [172], and, more recently, allogeneic CD70-targeting CAR-T cells (CTX130), which have recent published data from a phase I study (NCT04502446) [173]. In this first-in-human phase I trial of allogeneic CD70-targeting CAR-T cells for relapsed/refractory T cell lymphoma, 18 of 39 patients achieved an objective response, including 10 with PTCLs (4 CRs and 6 PRs). These results highlight encouraging activity in a population with few effective options. Safety signals included a case of dose-limiting toxicity in a PTCL-NOS patient who developed hemophagocytic lymphohistiocytosis and grade 4 cytokine release syndrome (CRS). Overall, CRS was the most common serious adverse event, occurring in 28% of patients. Notably, no graft-versus-host disease (GvHD) was observed, supporting the feasibility of healthy donor-derived CAR-T platforms. A separate phase I/II trial (NCT06492304) is currently evaluating another further modified allogeneic CD70-directed CAR-T product, CTX131, in relapsed/refractory hematologic malignancies [173].

Besides the use of allogeneic T cells, another promising approach is CD70-targeted CAR-NK cells derived from induced pluripotent stem cells (iPSCs), which may mitigate the risk of GvHD due to their lack of TCR expression and serve as an off-the-shelf therapeutic for patients. CD70 CAR-NK cells developed by Wang et al. have been gene-edited to knock out CD70 expression, thereby avoiding fratricide, and engineered to express a high-affinity, non-cleavable CD16 and an IL-15 receptor α/IL-15 fusion protein (IL15RF), which improves antibody-dependent cellular cytotoxicity and persistence [174]. These CD70 CAR-NK cells were able to eradicate T cell lymphoma cells both in vitro and in mouse xenograft models using the MT4 ATLL cell line and represent a promising novel approach [174]. A phase I dose escalation study of these CD70 CAR-NK cells for the treatment of relapsed/refractory TCL and AML is currently active (NCT06696846).

#### 3.2.7. CCR4

CCR4 is a chemokine receptor involved in T cell migration into the skin and is highly expressed on neoplastic T cells of CTCL (~62%) [175], ATLL (~90%) [176], and certain PTCL subtypes, including ALK-negative ALCL (~66.7%), PTCL-NOS (38%), and nTFHL-AI (34.8%) [177,178]. Higher CCR4 expression has also been correlated with disease severity [179,180]. CCR4 is a validated therapeutic target in TCL: mogamulizumab, a humanized, defucosylated anti-CCR4 monoclonal antibody (KW-0761) has received FDA approval for the treatment of *r/r* CTCL [181,182,183,184]. Despite initial activity with mogamulizumab, response durability is often limited [181,182,184,185]. Seeking to build on the achievements of mogamulizumab, CCR4-targeting CAR-T cells have been developed by Perera et al. [186] and Watanabe et al. [187]. In preclinical TCL models, Perera et al. demonstrated that CCR4-targeting CAR-T cells caused significant tumor lysis against multiple TCL cell lines, with superior efficacy observed in adult T cell leukemia (ATLL) and ALK-negative ALCL lines, concordant with CCR4 expression profiles in those diseases [186]. Significant tumor control was also achieved in ATLL murine xenografts treated with CCR4 CAR-T cells.

A further advantage of targeting CCR4 is the depletion of immunosuppressive Th2 cells and Tregs, enabling reactivation of endogenous anti-tumor responses that can act synergistically to improve outcomes [185,188]. This was demonstrated by Watanabe et al. [187], who showed that their CCR4 CAR-T product is enriched in Th1 cells due to fratricide of Th2 and Treg cells, possibly yielding improved anti-tumor efficacy and engraftment compared to CD19 CAR-T cells. However, Treg depletion may risk potentiating autoimmune adverse effects, which may prove a specific risk with CCR4-targeting CAR-T cells. The ongoing phase I trial of CCR4 CAR T cells for PTCL and CTCL (NCT07055477) should provide further insights into the safety profile of this agent.

#### 3.2.8. Other Targets in Preclinical Development

The development of CAR-T cell therapy for TCLs is rapidly evolving, with many other targets currently in preclinical development. These include CD3 [189], CD26 [190], B7-H3 [191], and CCR8 [192], CD37 [193], and CD56 [194].

Engineered cellular therapies directed against a range of PTCL targets have demonstrated proof-of-concept anti-tumor activity. However, particular challenges remain including fratricide, antigen overlap, and toxicity. Various innovations such as TRBC-restricted, CD30- or CCR4-targeted constructs and allogeneic gene-edited products are promising means of overcoming these issues. However, balancing potency, safety, and persistence while ensuring viable immune reconstitution represents the greater challenge of these agents in T cell malignancies compared to B cell lymphoma. The following section considers how novel multi-specific and antibody-based strategies might complement or substitute cell therapy in this space.

### 3.3. Bispecific Antibodies in T Cell Lymphoma

T cell-engaging bispecific antibodies have proven to be an invaluable development in cancer immunotherapy, offering an off-the-shelf strategy to redirect endogenous T or NK cells against tumors. They are an established modality in the B cell lymphoma armamentarium [195]. However, these agents are more challenging to deploy in a TCL context. As with CAR-T cells, targeting common T cell antigens that are simultaneously present on healthy T cells risks induction of T cell aplasia. Furthermore, therapies that induce effector T cell interaction with a T cell target can result in unintended T cell fratricide, as discussed in relation to CAR-T cell design [196]. Nonetheless, there is a growing portfolio of agents under development with promising early clinical data.

Leveraging the proven efficacy of the antibody–drug conjugate brentuximab vedotin, CD30 has emerged as an antigen of interest for bispecific antibody development. Circumventing the risk of fratricide, AFM13 is a first-in-class tetravalent bispecific antibody that binds CD30 and CD16a. CD16a is expressed on natural killer (NK) cells and macrophages, and AFM13 has been termed an innate cell engager (ICE) as a result. In preclinical studies, AFM13 achieved potent NK cell activation, enhancing cytokine production and killing CD30^+^ lymphoma targets in vitro. When combined with cytokine-preactivated peripheral blood or cord blood NK cells, AFM13 induced CAR-like activity with strong anti-tumor effects and improved survival outcomes in CD30^+^ lymphoma xenograft models [197].

An initial phase I study on AFM13 in heavily pre-treated patients with Hodgkin lymphoma demonstrated an ORR of 11.5%, though the disease control rate (defined as the sum of ORR and stable disease rate) was 61.5% [198]. Thereafter, a small phase Ib/II trial in patients with TCL with cutaneous involvement (CTCL or ALCL with cutaneous involvement) achieved an ORR of 40% (*n* = 14) [199]. Notably, responses to AFM13 monotherapy in HL were modest, and more substantial activity has been observed in combination with checkpoint inhibitors, where combination with pembrolizumab in *r/r* HL showed evidence of synergy with an ORR of 83%. Safety of the combination appeared comparable to either agent alone, and such a strategy could be adopted for PTCLs [200].

More recently, a phase II open-label study (REDIRECT) was performed in relapsed/refractory CD30+ PTCL. Of 108 patients treated on REDIRECT, the ORR was 32.4% (CR:10.2%), with a higher response rate seen in nTFHL-AI (53.3%). The median duration of response was 2.3 months, and the treatment proved tolerable, with no cases of cytokine release syndrome [201].

Given the mechanism of action, a new phase I study of AFM13 in combination with cord-blood-derived, cytokine-preactivated allogeneic NK cells has shown impressive efficacy in relapsed/refractory CD30+ lymphoma [202]. In this approach, NK cells are isolated from cryopreserved cord blood units, briefly preactivated with cytokines such as IL-12, IL-15, and IL-18 and expanded ex vivo using feeder cells before being precomplexed with AFM13 just prior to infusion. This generates an NK cell product that is highly enriched for CD56^+^/CD16^+^ effector cells. Patients received 2–4 cycles of lymphodepleting chemotherapy followed by infusion of the AFM13–NK product, along with weekly AFM13 dosing. Among 42 patients treated (37 with Hodgkin lymphoma and 5 with T cell lymphoma), the overall response rate was 92.9% and the complete response rate was 66.7%. At a median follow-up of 20 months, the 2-year event-free survival (EFS) and OS rates were 26.2% and 76.2%, respectively. Durable responses were evident, with 11 patients who had achieved CR maintaining this response for 14–40 months. No CRS or graft-versus-host disease occurred [202]. In this study, the trial population was dominantly composed of patients with Hodgkin lymphoma; of the patients with TCL, one died of unrelated causes and the other four all progressed. One patient achieved CR (duration ~ 12 months) and two achieved PR, with no response in the remaining two participants. Nonetheless, the TCL population had received a median of seven prior lines of therapy, representing a highly refractory cohort; it is therefore difficult to draw meaningful conclusions about the efficacy of this combination in the TCL context. A key advantage of this strategy is that cord blood-derived NK products do not require HLA matching and can be manufactured in advance, representing an “off-the-shelf” cellular immunotherapy, contrasting with the complex, bespoke production required for autologous CAR-T cells.

Responses to CD30/CD3 bispecific antibodies in Hodgkin lymphoma appear modest in the absence of checkpoint blockade, and consistent with this limited single-agent activity, Genmab has recently discontinued development of GEN3017 in relapsed/refractory CD30-positive lymphomas as part of a strategic pipeline reprioritization [203]. Hence, the future of these agents as monotherapies is uncertain.

Beyond CD30, several other bispecific antibodies are under development for TCL. Efforts to overcome the risk of T cell fratricide with TRBC1/2-targeting therapies continue but remain in the preclinical space [204].

GNC-038 is a first-in-class octavalent CD19/CD3/4-1BB/PD-L1 tetra-specific antibody, GNC-038. It functions as a T cell engager that can target both CD19 and PDL1. Accordingly, there are potential applications in both B cell and T cell malignancies, and this agent may counteract T cell-mediated inhibition based on its PD-L1-targeting capability. GNC-038 is being investigated in both acute lymphoblastic leukemia and lymphoma [205]. A further trial is underway specifically in NK/T cell lymphoma (NCT05627856).

ONO-4685 is a novel PD-1/CD3 bispecific antibody which has demonstrated preclinical activity against PD-1-expressing T cell malignancies, with dose-dependent granzyme-B-mediated killing in cell lines and in humanized mouse xenograft models [206]. A phase I trial in relapsed/refractory peripheral and cutaneous T cell lymphomas is now underway (NCT05079282). A key challenge in applying CD3-engaging bispecifics to PTCLs is the risk of engaging the malignant T cells themselves. In addition to triggering fratricide, this engagement could cause paradoxical tumor activation and major toxicities; the safety of this approach remains uncertain and must be carefully considered.

An intriguing variation on both conventional bispecific and genetically modified cellular therapies is the antibody-armed T cell (AATC). Unlike CAR-T cells, which require genetic modification and permanent receptor expression, autologous T cells can be “armed” ex vivo with a therapeutic bispecific antibody [207]. The arming is transient and produces effector cells that can immediately redirect cytotoxicity toward target tumor cells without genome editing or complex manufacturing. NCT05544968 is a first-in-human, phase I/II study of CD30 biAb-AATC, armed with a CD30/CD3 bispecific antibody. It is designed to treat relapsed/refractory CD30^+^ hematologic malignancies in pediatric and young-adult patients, with safety, feasibility, and early efficacy endpoints; the trial is scheduled to commence in late 2025.

### 3.4. Oncolytic Viruses

Oncolytic virus therapy is an emerging approach for cancer treatment. In 2015, the FDA approved T-Vec (talimogene laherparepvec), a second-generation oncolytic virus, for the treatment of melanoma [208]. The concept of oncolytic viruses originated from observations of tumor regression in patients following naturally acquired systemic viral infections. Oncolytic viruses, whether naturally occurring or genetically engineered, exert their anti-tumor effect by selectively replicating in cancer cells, stimulating host anti-tumor responses to cause immunogenic cell death [208,209].

The oncolytic effect of viral infection was highlighted in T cell lymphoma patients during the SARS-CoV-2 (COVID-19) pandemic. Three case reports have reported tumor regression after infection with the SARS-CoV-2 virus. One patient with MF achieved complete remission, while another with SS and a third with NK/T cell lymphoma experienced transient responses [210,211,212]. The virus likely exerted immunomodulatory properties through the induction of cytokine “storm”, which led to the reinvigoration of anti-tumor immunity in these patients.

Early clinical exploration of oncolytic virus therapy in T cell lymphoma dates to 2005, when Heinzerling et al. [213] evaluated the oncolytic effect of measles virus in five patients with CTCL. These patients received two cycles of therapy, with each cycle consisting of two intra-tumoral injections of live measles virus, followed by a subcutaneous injection of IFN-alfa 72 h and 24 h before each viral dose. Five of the six treated lesions (one patient received treatment for two lesions) showed tumor regression, with one of the treated lesions completely disappearing, highlighting the potential of this modality [213]. Consistent results were observed in the phase I study (NCT03017820) of a single dose of vesicular stomatitis virus (VSV) expressing interferon-β (IFN-β) with the sodium iodide symporter (NIS), where five out of seven patients with relapsed/refractory TCL demonstrated regression in one or more tumors, and three achieving a clinical response (two with PR and one with CR) [214]. Importantly, no dose-limiting toxicities were observed, CRS was limited to grade 1/2 [214].

More recently, preclinical studies of a rat protoparvovirus, H-1 (H-1PV), demonstrated that the virus can infect and stimulate oncolytic activity in CTCL cell lines (HH, HuT78, and MyLa) and CTCL tumor spheroids while sparing healthy T cells and PBMCs [215]. Although most data regarding oncolytic viruses in TCL is derived from the CTCL context, these early findings suggest potential applicability in patients with PTCL. Three clinical trials of oncolytic viruses involving PTCL patients are currently active (NCT06508463, NCT05387226, NCT03017820).

There is significant potential for combining oncolytic viruses with orthogonal immunotherapeutic modalities. Numerous studies have demonstrated their capacity to reinvigorate antitumor immunity by remodeling the tumor microenvironment (TME) from an immune-suppressed “cold” state to an inflamed “hot” state [209]. This immunologic shift may enhance responsiveness to immune checkpoint inhibitors, CAR-T cell therapy, and bispecific T cell engagers. Combining oncolytic viruses with ICI immunotherapy has been evaluated in advanced melanoma, where adding an oncolytic virus to ipilimumab (anti-CTLA-4) improved response rates compared with ipilimumab alone (39% vs. 18%) [216]. Such combinations have not been investigated in patients with TCL but represent an intriguing area for future investigation.

### 3.5. Immunomodulatory Imide Drugs (IMiDs)

Immunomodulatory imide drugs (IMiDs) are structural analogues of thalidomide with potent anti-cancer and immunomodulatory properties [217]. The class includes thalidomide, lenalidomide, and pomalidomide. These drugs not only exhibit direct cytotoxicity against tumor cells but can also modulate the TME via their anti-angiogenic effects, T cell co-stimulation, and enhancing antibody-dependent cellular cytotoxicity (ADCC) of NK cells [217,218].

The use of lenalidomide as monotherapy in PTCL has been explored in multiple phase II trials across various disease subtypes, where it has shown a tolerable safety profile and comparable efficacy, with ORRs of 22–30%, respectively [219,220,221]. Response rates differ by disease subtype, with nTFHL-AI (ORR 31%) [219] and ATLL patients (ORR 42%) [222], responding better to lenalidomide as monotherapy. These results align with case reports in nTFHL-AI with refractory disease, where single-agent thalidomide has successfully induced a treatment response, with one patient achieving complete remission [223,224]. In these studies, the investigators demonstrated that thalidomide therapeutically modulates the tumor microenvironment in nTFHL-AI, reducing EBV-driven proliferation of monoclonal B cells, which likely contribute to the pro-tumorigenic TME [223].

Whilst IMiDs as a single agent were able to induce clinical response, PTCL patients do eventually relapse, with a progression-free survival of approximately 4 months [220,222]. Subsequent studies have investigated the utility of combining IMiDs with multi-agent chemotherapy regimens. In PTCL, the addition of thalidomide to standard CHOP (cyclophosphamide, adriamycin, vincristine, and prednisone) improved CR rates (mixed frontline and relapsed patient cohort) from 36.4% in the control (chemotherapy alone) group to 50% in the combination group [225]. Encouragingly, a phase II trial of an all-oral regimen of chidamide plus prednisone, cyclophosphamide, and thalidomide (CPCT) for *r/r* PTCL patients achieved an ORR of 71.1% (32/45) and a 28.9% CR rate, with a PFS of 8.5 months [226]. The clinical utility of IMiDS in first-line treatment has also been demonstrated in a trial comparing standard CHOP therapy versus GDPT (gemcitabine, cisplatin, prednisone, thalidomide) in newly diagnosed PTCL patients, where the ORR and CR rates of the GDPT group were significantly superior to the CHOP group (ORR 66.3% vs. 50% *p* = 0.042, CR 42.9% vs. 27.6% *p* = 0.049) [227].

Due to their immune-modulating properties, IMiDs have been investigated in combination with immunotherapies. In CTCL, a phase II trial of the combination of anti-PD-L1 and lenalidomide showed a significantly better ORR compared to durvalumab alone (58% vs. 36%) [228]. While this combination has not been investigated in PTCLs, a few studies have shown possible synergistic effects that may offer benefits to PTCL patients as well. In a small series of three *r/r* ENKTL patients, an anti-PD1 antibody combined with chidamide, etoposide, and thalidomide led to two CRs and one PR, suggesting a possible synergistic effect of IMiDs with immunotherapies [114]. However, enhanced efficacy may be accompanied by increased toxicity, as in the phase III trial of IMiD-anti-PD1 combination treatment in multiple myeloma, where a higher frequency of serious adverse events and treatment-related deaths resulted in termination of these studies [229,230]. Nevertheless, IMiDs have been shown to synergize with monoclonal antibody treatment in a B cell lymphoma context [231]. This is likely due to their ability to enhance NK-mediated ADCC and consequently could be investigated in combination with monoclonal antibodies or ADCs such as mogamulizumab (anti-CCR4) and brentuximab vedotin (anti-CD30) in *r/r* PTCL patients. In a phase I dose escalation study, the combination of lenalidomide with brentuximab vedotin showed promising responses, with two out of six patients achieving CRs and three patients achieving PRs (NCT03302728) [232]. Additionally, a phase II trial examining the same combination (NCT03409432) showed an ORR of 27.8% for CTCL (2 of 18 CRs) and 50% for PTCL (3 of 8 CRs) [233]. As previously mentioned, multiple clinical trials are investigating lenalidomide in combination with anti-PD1 (NCT04231370, NCT05182957, NCT01919619), anti-PD1 and chemotherapy (NCT04040491, NCT04038411), and anti-PDL1 (NCT03011814, NCT03054532). Besides enhancing NK cell activity, IMiDs are also known to provide co-stimulation to T cells, polarize the TME into Th1, and promote T cell survival [217], underscoring the rationale for combination with T cell-based cancer immunotherapies like CAR-T cell therapy and bispecific antibodies. Currently, the combination of IMiDs with CAR-T cell therapy or bispecifics is being trialed in multiple myeloma [218], and if successful, it could prompt consideration of combination therapy in TCL patients as well.

### 3.6. Other Agents

#### 3.6.1. Denileukin Diftitox

Denileukin diftitox is a recombinant fusion protein in which human interleukin-2 is conjoined to diphtheria toxin fragments A/B, delivering the toxin into cells that express the high-affinity IL-2 receptor (CD25). Once bound to CD25, the complex is internalized and the diphtheria toxin broadly arrests protein synthesis, leading to cell death. As discussed with CCR4-targeting therapies, denileukin diftitox also depletes CD25^+^ regulatory T cells, offering a dual rationale though modulation of the TME and augmentation of tumor immune responses.

A pivotal phase III trial in relapsed/refractory CD25^+^ CTCL demonstrated an ORR of 44% (CR 34%), with a median response duration of 6.9 months. Median progression-free survival was significantly longer (over 2 years) in the denileukin arms versus ~124 days in placebo. Toxicities included infusion reactions, elevated liver enzymes, hypoalbuminemia, and the characteristic capillary leak syndrome [234]. The original formulation was withdrawn in 2014 for manufacturing reasons, but an improved-purity version (CXDL) has since been developed.

The registrational phase III of denileukin diftitox CXDL in relapsed/refractory CTCL reported an ORR of 36.2% (CR 8.7%) and median DOR of 8.9 months, leading to FDA approval in 2024 [235]. Smaller phase II studies in PTCL have also shown single-agent activity, with ORR around 40–50% (particularly in CD25^+^ cases), and early efforts have combined the drug with CHOP in frontline PTCL, establishing feasibility.

The CONCEPT trial evaluated the addition of denileukin diftitox to CHOP in previously untreated PTCL. In this phase II study (*n* = 49), the overall response rate was 65%, with complete responses in 45% of patients. At a median follow-up of 25.6 months, the median DOR was approximately 30 months, median PFS was 12 months, and median OS had not been reached, with an estimated OS rate of 63% at last analysis [236]. These data suggest that a subset of patients achieved durable remissions; however, no longer-term (>5 year) follow-up of this cohort has been reported.

Denileukin diftitox is a clinically validated option in CTCL, with potential applications in PTCL, though careful monitoring for capillary leak and hepatic toxicity is required, and longer-term outcome data would be valuable.

#### 3.6.2. Brentuximab Vedotin

The antibody–drug conjugate brentuximab vedotin (BV) combines a CD30 monoclonal antibody with the antimitotic agent monomethyl auristatin E. Phase I/II studies suggested clinical utility in TCL, with the most pronounced benefits observed in patients with CTCL and systemic ALCL (sALCL) in these early trials [237,238,239]. In the phase II trial of BV patients with sALCL, the ORR was 86%, with a CR rate of 57% and a median duration of response of 12.6 months [238]. Long-term follow-up at five years confirmed that this treatment was able to deliver durable disease control to a substantial number of patients, with median overall survival (OS) not reached. In the 38 patients who achieved a CR, neither median OS nor PFS were reached. Sixteen patients of this group had not required additional therapy apart from a consolidative auSCT, with eight patients being auSCT recipients and another eight patients not receiving auSCT or any further anti-cancer treatment after conclusion of study participation [240].

By way of contrast, the phase II data for brentuximab vedotin in patients with relapsed/refractory PTCL reported by Horwitz et al. demonstrated substantially reduced efficacy in histologic subtypes other than sALCL. ORR was 41%, and CR was observed in 8 of 35 participants (22%); however, median PFS was a short 6.7 months in patients with nTFHL-AI and only 1.6 months in patients with PTCL-NOS [241].

Combination strategies have also been explored in PTCL, most notably the phase III ECHELON 2 trial, which compared BV in combination with the cytotoxic backbone of cyclophosphamide, doxorubicin, and prednisolone (BV-CHP) against the standard-of-care CHOP (cyclophosphamide, doxorubicine, vincristine, and prednisolone) in patients with newly diagnosed CD30-positive PTCL. In this study of 452 patients, of whom 70% had sALCL, 3-year OS was 77% versus 69% (HR 0.66, *p* = 0.02), with median PFS of 48.2 months versus 20.8 months (HR 0.71, *p* = 0.01) [158]. At 5 years of follow-up, these results were sustained, with estimated 5-year PFS of 51% for BV-CHP compared to 43% for CHOP. Estimated 5-year OS was 70% and 61%, respectively [242].

While CTCL is not the predominant focus of this review, it is worth noting the results of the phase III ALCANZA study in patients with CD30-positive relapsed or refractory CTCL, in whom BV was compared to physician’s choice of either oral methotrexate or bexarotene. A total of 128 patients were included. Outcome data favored BV treatment, with an objective response lasting 4 or more months (ORR4) being 54.7% in the BV group versus 12.5% in the physician’s choice group (*p* < 0.001), and ORR of 65.6% versus 20.3% (*p* < 0.001). The CR rate was 17.2% versus 1.6% (*p* = 0.002). Median PFS was 16.7 months for BV-treated patients versus 3.5 months for the physician’s choice cohort (HR 0.38, *p*= 0.001), with a median follow-up of 36.8 months [157]. The final analysis of the data at a median follow-up of 45.9 months continued to demonstrate clinical benefit from BV treatment in this group of patients [243].

#### 3.6.3. Lacutamab

Lacutamab, also known as IPH4102, is a fully humanized monoclonal antibody targeting KIR3DL2, an inhibitory immune receptor usually expressed on small numbers of CD8-positive T cells and NK cells in health which is upregulated and highly expressed in selected subtypes of CTCL [244,245,246,247,248]. Assessment of the mode of action of lacutamab in preclinical work in CTCL suggested a highly tumor-specific effect causing antibody-dependent cell death and phagocytosis, with reduced tumor growth and prolonged survival in animal models [244,249]. Phase I–II trials confirmed clinical benefit with good tolerance in patients with SS and MF, with long-term results from the phase II study in SS recently reported showing ORR of 42.9% and median duration of response of 25.6 months (range, 11—not evaluable) [250,251,252].

Later research confirmed that KIR3DL2 is also upregulated in certain PTCL subtypes when assessed in vivo by either flow cytometric methods or immunohistochemistry, with highest rates of expression observed in PTCL-NOS and AITL [253]. Preclinical work in PTCL suggested a combinational effect on cell death in PTCL in tumor models when lacutamab was used with cytotoxic agents, either CHOP or pralatrexate [254].

Based on encouraging results for lacutamab in CTCL and bolstered by the preclinical data in PTCL, a phase Ib trial (NCT05321147) was developed to explore the efficacy and safety of lacutamab monotherapy in relapsed/refractory PTCL [255]. However, interim results from this study suggested a lack of sufficient efficacy, which in early 2024 led to discontinuation of the PTCL monotherapy development pathway, with a refocus predominantly on lacutamab monotherapy in CTCL and combination strategies in PTCL [256]. The phase II randomized non-comparative clinical trial of lacutamab in combination with gemcitabine and oxaliplatin (GemOx) versus GemOx alone (KILT study; NCT04984837) is ongoing in patients with *r/r* PTCL [257].

#### 3.6.4. Mogamulizumab

Mogamulizumab, a CCR4 monoclonal antibody, has demonstrable clinical benefit in CTCL, particularly in advanced disease with a leukemic component. The phase III MAVORIC study compared mogamulizumab in comparison to the HDACi–vorinostat in patients with *r/r* MF and SS, stratified according to stage. Median PFS was 7.7 months for mogamulizumab versus 3.1 months for vorinostat (HR 0.53, 95% CI 0.41–0.69, *p* < 0.0001), with similar rates of adverse events between both groups. For patients with SS, HR was 0.32 (95% CI, 0.21–0.49) [182]. Improved ORR and time to next treatment (TTNT) appeared to correlate to the degree of blood involvement, with ORR among patients with a high burden of Sezary cells being 37.4% for mogamulizumab-treated patients versus 3.2% for vorinostat-treated patients (*p* < 0.0001) [258].

An optimal therapeutic niche for mogamulizumab in patients with PTCL is still being delineated. Phase II data from selected studies suggested an ORR between 11.4 and 34%, although the latter study included both PTCL and CTCL patients [185,259]. Post marketing surveillance data from Ishitsuka et al., Japan, reported an ORR of 34.5% in their PTCL cohort, with highest responses in patients with nTFHL-AI (50%) [260]. Durable responses are not guaranteed, as noted elsewhere in this review.

It is important to note, however, that there seems to be a specific clinical benefit to the use of mogamulizumab in patients with ATLL, particularly in the setting of relapsed/refractory disease. Data is predominantly drawn from small case series and selected phase II studies at present, but the agent appears active and well tolerated either given as monotherapy or in combination, and larger-scale trials are key [261,262,263,264,265].

#### 3.6.5. MEDI-570

As elucidated earlier in this review, the high expression of the inducible T cell costimulator (ICOS) on Tfh, coupled with an evolving understanding of the role ICOS in lymphomagenesis, led to the development of clinical trials of MEDI-570, a human afucosylated, IgG kappa monoclonal anibody targeting ICOS. Initial trials in the autoimmune disorder systemic lupus erythematosus (NCT01127321) were terminated due to emergent side effects deemed unacceptable in the setting of a chronic inflammatory disease [266]; however, the observation of a significant reduction in memory CD4-positive ICOS-positive cells in the study population refocused interest in the utility of this agent in nTFHL. The phase I dose escalation study (NCT02520791) explored intravenous administration of MEDI-570 once every three weeks in a cohort of patients with relapsed/refractory PTCL. Twenty-three patients were included, including eighteen in the dose escalation phase, with patients having received a median of three prior lines of treatment. Of these, 17 had nTFHL (nTFHL-AI in 16 patients, follicular TFHL in 1). No DLTs were observed. Treatment-emergent adverse events (TEAEs) included predominantly grade 1–2 side effects, including infusion-related reactions (IRRs) (48% of the patients), fatigue (35% of the patients), and nausea (30% of the patients), while grade 3–4 TEAEs were predominantly reduced CD4 counts (57% of the patients), lymphopenia, and anemia, as well as a grade 3 IRR in one patient leading to augmented mandatory premedication for subsequent subjects. ORR was 30% (7/23 patients), with 2 patients achieving a CR and 5 achieving PR. All responding patients had nTFHL-AI, with ORR in patients with this subtype being 44%. Median PFS for nTFHL-AI patients was 2.9 months (95% CI: 1.9–6.7 months), while median OS was 17.1 months [267]. Further study is needed to identify the optimal therapeutic niche for this agent, noting also the potential for rational combinations.

**Table 2:** Studies evaluating cellular therapies, oncolytic viruses, and immunomodulatory agents in peripheral T cell lymphoma, organized by therapeutic approach and target.

Regimen	Phase	Trial	Prior Lines of Therapy	No of Patients (TCL Cohort)	ORR	CR	Median PFS	OS	Reference
CAR-T Cell Therapies									
CD4-specific CAR-T	Phase I	NCT04162340	N/A	(Preliminary data) Total: 3 SS: 1 Transformed MF: 1 AITL: 1	100% (2 CR, 1 PR)	66% (2 out of 3)	N/A	N/A	[130]
	Phase I	NCT04712864	Active, Not Recruiting						
	Phase I	NCT03829540	Recruiting						
CD5-specific CAR-T	Phase I	NCT03081910 (MAGENTA)	Median: 5 lines (Range 2–18)	Total: 9 MF/SS: 1 CTCL: 1 AITL: 2 PTCL: 4 ATLL: 1	44% (2 CRs, 1 PR, 1 mixed radiographic response)	22% (2 out of 9)	N/A	N/A	[137]
	Phase I	NCT04767308	(Range 2–6)	(Preliminary data) Total: 3 AITL: 2 SPTCL: 1	100% (1 CR, 2 PR)	33% (1 out of 3)	N/A	N/A	[136]
	Phase I	NCT04594135	Unknown status						Preclinical and preliminary clinical data of 1 patient [135]
	Phase I	NCT06633341	Recruiting						
	Phase I	NCT07022964	Recruiting						
CD7-specific CAR-T	Phase I	NCT04928105	Median: 6 (Range: 2–12)	(Preliminary data) Total: 5 PTCL-NOS: 2 MEITL: 1 HSTCL: 1 NKTCL: 1	80% (3 CR, 1 PR)	60% (3 out of 5)	N/A	N/A	[148]
	Phase I	NCT05377827	N/A	(Preliminary data) Total: 5 PTCL:1 T-PLL:2 Gamma-delta TCL: 2	80% (2 CR, 2 PR, 1 SD)	40% (2 out of 5)	N/A	N/A	[268]
	Phase I	NCT04004637	Unknown status						Preliminary data on T-ALL/T-LBL [269]
	Phase I	NCT05290155	Completed, results not published						
	Phase II	NCT05059912	Unknown status						
	Phase I/II	NCT06925464	Recruiting						
	Phase I	NCT05979792	Not yet recruiting						
	Phase I	NCT04480788	Unknown status						
	Phase I	NCT04264078	Unknown status						
	Phase I	NCT04934774	Unknown status						
	Phase I	NCT04823091	Recruiting						
	Phase I	NCT05995028	Recruiting						
	N/A	NCT07008872	Not yet recruiting						
	N/A	NCT05620680	Recruiting						
TRBC1-specific CAR-T	Phase I/II	NCT03590574 (AUTO4)	Median: 2 (Range 1–5)	Total: 10 AITL: 4 PTCL-NOS: 5 ALCL: 1	66.6% (6 of 9)	44.4% (4 of 9)	Median 4.7 months	Median OS was not reached	[270]
	Phase I	NCT04828174	Trial suspended						
CD30-specific CAR-T	Phase I	NCT01316146	N/A	Total: 9 Hodgkin’s lymphoma: 7 ALCL: 2	33.3% (3 of 9)	33.3% (3 of 9)	N/A	N/A	[169]
	Phase I	ChiCTR-OPN-16009069	N/A	Total: 9 Hodgkin’s lymphoma: 6 ALCL: 3	77.7% (7 CR)	77.7%	Median 13 months		[170]
	Phase I	NCT04526834	Active, not recruiting						
	Phase I	NCT07048353	Not yet recruiting						
	Phase I	NCT05208853	Unknown status						
	Phase I	NCT02917083 (RELY-30)	Recruiting						
	Phase I	NCT04653649	Unknown status						
	Phase II	NCT04083495	Recruiting						
	Phase I	NCT06494371	Recruiting						
Allogeneic CD30 CAR-EBVST cells	Phase I	NCT04288726	Recruiting						
Allogeneic CD30 CAR-EBVST cells with constitutive IL7R (C7R)	Phase I	NCT06176690	Not yet recruiting						
CCR4-expressing CD30-specific CAR-T cells	Phase I	NCT03602157	Recruiting						
CD30-specific CAR-T as consolidation after BEAM and autologous HSCT	Phase I	NCT02663297	83% of patients have one line of salvage therapy before autologous HSCT, 17% required second line of therapy	Total: 21 Hodgkin’s lymphoma: 11 ALCL: 4 AITL: 1 PTCL-NOS: 1 Grey zone lymphoma: 1	At median follow-up of 48.2 months, 5 patients with T cell lymphoma have died	N/A	Median 32.3 months	Not reached	[171]
Allogeneic CD70-specific CAR-T cells	Phase I	NCT04502446 (COBALT-LYM)	Median: 2.5 for PTCL, 5 for CTCL	Total: 39 PTCL: 22 (9 ATLL, 8 PTCL-NOS, 4 AITL, 1 ALCL) CTCL: 17	46.2% (18 of 39)	19.4% (6 of 39)	N/A	N/A	[179]
	Phase I/II	NCT06492304	Recruiting						
CCR4-specific CAR-T cells	Phase I	NCT07055477	Recruiting						
CD37-specific CAR-T cells	Phase I	NCT04136275	Median 6 (range 3–8)	Total: 5 Double-hit HGBCL: 2 CTCL:1 Hodgkin’s lymphoma: 1 NKTCL: 1	80% (3 CR)	60% (3 of 5)	N/A	N/A	[193]
CD56-specific CAR-T	Phase II	NCT05941156	Recruiting						
CAR-NK Cell Therapies									
CD5-specific CAR-NK	Phase I	NCT06909474	Recruiting						
CD70-specific CAR-NK cells	Phase I	NCT06696846	Not yet recruiting						
Oncolytic Virus									
Oncolytic Virus Injection (RT-01)	Phase I	NCT06508463	Unknown status						
Vesicular Stomatitis Virus (VSV)	Phase I	NCT05387226	Recruiting						
Vesicular Stomatitis Virus (VSV)	Phase I	NCT03017820	Recruiting						
Single Agent Immunomodulatory Drugs (IMiDs)									
Lenalidomide Monotherapy	Phase II	NCT01724177	Median: 2 (Range 1–4)	Total: 26 (All ATLL)	42%	19.2% (4 CR and 1 unconfirmed)	3.8 months	20.3 months	[228]
Lenalidomide Monotherapy	Phase II	NCT00322985	Median: 1 (range 0–5)	Total: 40	26% (10 of 39)	8%	4 months	12 months	[226]
Lenalidomide Monotherapy	Phase I	NCT01169298	Median: 1 (Range 1–3)	Total: 13 ATLL: 9 Other PTCL: 4	36% (4 of 11 evaluable)	0%	3.4 months	N/A	[271]
Lenalidomide Monotherapy	Phase II	NCT00655668 (EXPECT)	Median: 3 (Range 1–11)	Total: 54 AITL: 26 PTCL-NOS: 20 CTCL (MF): 3 sALCL: 3 pcALCL:1 ENKTL: 1	22% (12 of 54)	11%	2.5 months	N/A	[227]
Lenalidomide Monotherapy	Phase II	NCT01036399	Median: 4 (Range 2–7)	Total: 10 (All PTCL-NOS)	30%	30% (3 of 10)	N/A	N/A	[225]
Immunomodulatory Drugs (IMiDs) + Chemotherapy									
Chidamide plus prednisone, cyclophosphamide, and thalidomide (CPCT)	Phase II	NCT02879526	At least 1 prior line	Total: 45 AITL: 20 PTCL-NOS: 17 Other subtypes: 8	71.1% (32 of 45)	28.9% (13 of 45)	8.5 months	17.2 months	[232]
CHOP (cyclophosphamide, doxorubicin, vincristine, prednisone) vs. GDPT (gemcitabine, cisplatin, prednisone, thalidomide) in newly diagnosed PTCL patient	Phase IV	NCT01664975	0 (newly diagnosed cohort)	Total: 153 PTCL-NOS: 31 AITL: 37 ALCL: 49 Other subtypes: 36	66.3% (GDPT) versus 50.0% (CHOP)	42.9% (GDPT) vs. 27.6% (CHOP)	4-year PFS: 63.6% (GDPT) vs. 53.0% (CHOP)	4-year OS: 66.8% (GDPT) vs. 53.6% (CHOP	[233]
Lenalidomide in combination with vorinostat and dexamethasone	Phase I/II	NCT00972842	Median: 1 (Range 1–2)	Total: 8 AITL: 5 PTCL-NOS:1 ALCL: 1	25% (2 of 7 evaluable)	14.2% (1 of 7)	2.2 months	6.7 months	[272]
CHOP alone vs. CHOP plus thalidomide	N/A	N/A	N/A	Total: 46 NKTCL: 21 PTCL: 9 ALCL: 7 AITL: 4 Other subtypes: 5	79.2% (thalidomide group) vs. 63.6% (CHOP alone)	50% (thalidomide group) vs. 36.4% (CHOP alone)	12 months (thalidomide group) vs. 6 months in CHOP alone	Undefined (thalidomide group) vs. 17 months in CHOP alone	[231]
Romidepsin, 5-azacitidine, Dexamethasone, plus Lenalidomide	Phase I	NCT04447027	Median: 2 (Range 1–8)	Total: 26 Nodal TCL: 16 MF: 6 ATLL: 4	56% (25 evaluable)	12%	1-year PFS: 14.9%	1-year OS: 63.3%	[273]
Lenalidomide plus CHOEP	Phase I/II	NCT02561273	0 (newly diagnosed)	Total: 39 PTCL-NOS: 19 AITL: 16 ALCL: 3	69%	49%	2-year PFS: 55%	2-year OS: 78%	[274]
Romidepsin and Lenalidomide	Phase II	NCT02232516	0 (newly diagnosed)	Total: 29 AITL: 16 PTCL-NOS: 10 ATLL: 2 EATL: 1	65.2%	26.1%	2-year PFS: 31.5%	2-year OS: 49.5%	[275]
Lenalidomide plus Gemcitabine	Phase I/II	NCT05105412	Terminated						
Bendamustine Combined With Chidamide and Lenalidomide	N/A	NCT07072221	Recruiting						
Chidamide Combination with Lenalidomide	Phase II	NCT04329130	Unknown status						
lenalidomide plus CHOP (L-CHOP) versus CHOP alone	Phase II	NCT04922567	Recruiting						
CHOP plus Lenalidomide	Phase II	NCT01553786	Completed						
Immunomodulatory Drugs (IMiDs) + Immunotherapy									
Lenalidomide + Sintilimab (anti-PD1)	Phase II	NCT04231370	Unknown status						
Anti-PD1 + Lenalidomide and azacytidine	Phase II	NCT05182957	Unknown status						
Lenalidomide, anti-PD1, Chidamide and Gemcitabine	Phase IV	NCT04040491	Unknown status						
Lenalidomide, anti-PD1, Chidamide and Etoposide	Phase IV	NCT04038411	Unknown status						
Durvalumab (anti-PDL1) with or without lenalidomide	Phase I/2	NCT03011814	Active, not recruiting						
	Phase II	NCT03054532	Unknown status						
Lenalidomide plus Brentuximab Vedotin	Phase I	NCT03302728	Completed						
	Phase II	NCT03409432	Completed						
Lenalidomide and Ipilimumab after Stem cell transplant	Phase II	NCT01919619	Completed						

Abbreviations: AITL, angioimmunoblastic T cell lymphoma; ALCL, anaplastic large cell lymphoma; ALK, anaplastic lymphoma kinase; ALK-, ALK-negative; ALK+, ALK-positive; ASCT, autologous stem cell transplantation; CR, complete response; DOR, duration of response; EATL, enteropathy-associated T cell lymphoma; ENKTL, extranodal natural killer/T cell lymphoma; HSTCL, hepatosplenic gamma delta T cell lymphoma; MEITL, monomorphic epitheliotropic intestinal T cell lymphoma; MF, mycosis fungoides; N/A, not applicable; NR, not reached; ORR, overall response rate; OS, overall survival; PCET, PD-1 antibody + chidamide + etoposide + thalidomide; PD, progressive disease; PD-L1, programmed death-ligand 1; PFS, progression-free survival; PTCL, peripheral T cell lymphoma; PTCL NOS, peripheral T cell lymphoma not otherwise specified; TCL, T cell lymphoma; TFH, T-follicular helper.

**Figure 3:**
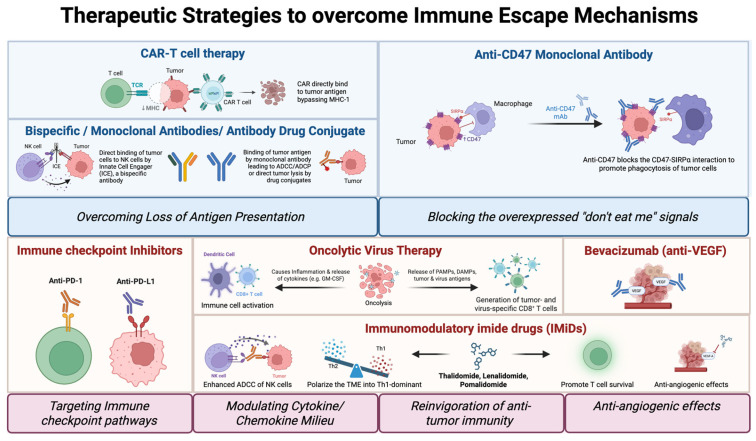
Therapeutic strategies to overcome immune escape mechanisms in peripheral T cell lymphomas. Approaches include CAR-T and CAR-NK cell therapies, bispecific and antibody–drug conjugates that overcome loss of antigen presentation; anti-CD47 antibodies that block “don’t-eat-me” signals; checkpoint inhibitors and oncolytic viruses that modulate tumor–immune interactions; and immunomodulatory agents that reshape the cytokine milieu and promote T cell activation. Together, these strategies exemplify the convergence of cellular, molecular, and immunologic modalities toward personalized immunotherapy in PTCL.

Collectively, these emerging immunotherapeutic modalities demonstrate the growing capacity to target both tumor-intrinsic and microenvironmental mechanisms of immune escape in PTCL. Checkpoint inhibitors, engineered cellular therapies, bispecific antibodies, antibody–drug conjugates, and immunomodulatory agents act through complementary mechanisms that together broaden the therapeutic landscape. Figure 3 summarizes these strategies and the diverse immunologic axes they engage to restore effective anti-tumor immunity. Though exciting, future research must seek to define which patient subgroups will benefit most from which therapy and identify rational approaches to therapy combination, sequencing, and monitoring.

## 4. Biomarkers for Informing Management of PTCL

The heterogeneity of PTCL underscores the urgent need for robust biomarkers that can guide therapeutic choice and predict response to emerging immunotherapies. As discussed in the checkpoint inhibitor section, most of the biomarker literature to date comes from studies of PD-1/PD-L1 blockade, often extrapolated from non-PTCL contexts, and includes factors such as PD-L1 expression, EBV status, tumor mutational burden, and 9p24.1 structural variants. While these data provide important proof-of-concept, they remain incomplete and inconsistently predictive in PTCL. Moving beyond immune checkpoint therapy, additional modalities are beginning to yield important insights, including imaging biomarkers such as FDG-PET, genomic and circulating tumor DNA markers, immunohistochemical correlates, and gene expression-defined subtypes. Integrating these data into a coherent framework, though nascent, is essential for biomarker-driven stratification and personalized therapeutic strategies in PTCL.

### 4.1. Positron Emission Tomography

Functional imaging with 18F-FDG PET/CT is a cornerstone of response assessment across PTCLs. Baseline metabolic tumor volume, response kinetics during treatment, and end of treatment response all have prognostic implications in PTCLs [276,277,278]. Novel radiotracers targeting immune cell populations in an attempt to directly visualize the tumor microenvironment hold promise as potential biomarkers in PTCLs. For example, PD-1/PD-L1 axis radiotracers are currently under investigation as predictors of response to immune checkpoint inhibitor therapy, but data in PTCLs is currently lacking [279].

### 4.2. Genomics

Recent reports suggest that high pre-treatment circulating tumor DNA (ctDNA) levels predict lower response and survival rates in T and NK cell lymphomas [268]. In addition, the monitoring of somatic mutations over time has utility for predicting relapse [268,280]. Mutations of *RHOA*, *IDH2*, *TET2*, and *DNMT3A* are highly recurrent in nTFHL-AI and Tfh cell lymphomas and aid in diagnosis [270]. In nTFHL-AI particularly, *RHOA* and *IDH2* show promise as MRD markers because they are not present in pre-lymphoma clones [281]. The vast majority of patients reported by Kim et al. and Yannakou et al. were treated with conventional chemotherapy approaches as opposed to immunotherapies, however [280,281]. In a study of ctDNA in nTFHL-AI by Zhang et al., RHOA G17 codon mutations were significantly associated with genes involved in “PD-L1 expression and PD-1 checkpoint pathway in cancer” by pathway analysis, suggesting that patients harboring such mutations may respond more favorably to checkpoint inhibition [271].

### 4.3. Immunohistochemistry

CD30 is a key biomarker for treatment selection in the setting of PTCLs, as some degree of CD30 expression is required to justify treatment with brentuximab vedotin. In the ECHELON-2 study, a CD30 cut-off of 10% was used for patient eligibility, but there was no correlation between CD30 expression and clinical outcome [242].

ALK positivity is a key determinant of outcome in systemic ALCL; however, this does not predict response rates or survival outcomes post brentuximab vedotin in either the first-line or the relapsed setting [240,242]. The presence or absence of DUSP22 and TP63 rearrangements in ALK-negative ALCL is known to stratify patients by clinical outcome, with DUSP22 rearrangements having a positive impact and TP63 rearrangements having a negative impact on prognosis [272]. The utility of DUSP22 and TP63 rearrangements as biomarkers of response to BV is currently unknown given the low number of BV-treated patients described in the literature to date.

In ATLL, CCR4 mutations and immunohistochemistry (which is inversely correlated with mutation status) have been associated with superior mogamulizumab effect [273,274]. However, other reports assessing mogamulizumab for the treatment of T cell lymphoma have not demonstrated this correlation between CCR4 expression and clinical outcome [185].

### 4.4. Gene Expression

Huang et al. defined various molecular subtypes of PTCL using an integrated genomic and transcriptomic approach [11]. The “Tfh-like” subtype expressed higher levels of CRBN (Cereblon), TIGIT, and CD52, while increased expression of immune checkpoint genes such as PD-L1 and LAG3 was found in the “inflammatory” subtype. The “mesenchymal” subtype expressed higher levels of TNFRSF8 (CD30), IDO1, and TIM-3. Du et al. used spatial transcriptomics to show that CCL17 and CCL22, which encode the dominant ligands for CCR4, are upregulated within the tumor microenvironment of nTFHL-AI, suggesting that anti-CCR4 therapies such as mogamulizumab may have activity in this disease [275]. Such approaches provide tantalizing insights into how the heterogeneity of PTCL can be teased apart and provides a rationale for a more personalized approach to immunotherapy selection. However, at this time, correlation with clinical outcomes is lacking and further investigation is thus required.

### 4.5. Integrating Biomarkers

Collectively, these biomarker studies demonstrate both the progress and limitations of biomarker discovery in PTCL. As discussed in Section 3.1, PD-L1 expression and 9p24.1 amplification remain the best studied correlates of immune checkpoint sensitivity, but their predictive power is inconsistent across subtypes. EBV positivity may confer distinct immune–antigenic contexts relevant to both tumor biology and immunotherapy response, while TRBC restriction defines a potential avenue for selective CAR-T targeting. Quantitative and longitudinal ctDNA profiling offers a dynamic measure of disease burden and molecular response, and preliminary data suggest that circulating mutations such as RHOA G17V could enrich for immunotherapy-responsive phenotypes. TMB has been evaluated as a surrogate of neoantigenicity, but levels are typically low in PTCL and have not consistently correlated with response. Integration of these orthogonal biomarkers—genomic, transcriptomic, serologic, and microenvironmental—into cohesive, multi-omic frameworks will be critical to translate biological insight into clinically actionable stratification. However, existing approaches remain exploratory and lack prospective validation, underscoring the need for harmonized assays and correlative biomarker programs embedded within future clinical trials. At present, the clinical utility of these biomarkers remains limited, either individually or cumulatively considered.

## 5. Perspectives and Outlook on Immunotherapy in PTCL

Immunotherapy in PTCL remains an emerging frontier modality rather than an evidence-based standard. While isolated signals of activity have been observed across immune checkpoint blockade, antibody–drug conjugates, bispecific antibodies, and other immune-modulatory agents, these approaches are yet to consolidate into a coherent therapeutic framework. The absence of biomarker-directed patient selection and limited understanding of the tumor–microenvironment interface—particularly the immunologic consequences of key genomic and epigenomic alterations—continue to hinder rational treatment design. Without this biological foundation, it remains difficult to define evidence-based therapeutic sequencing strategies or identify synergistic combinations with cytotoxic, epigenetic, or targeted agents. These uncertainties are compounded by the intrinsic heterogeneity of PTCL, especially within PTCL-NOS, where diverse cell-of-origin and transcriptional states dilute statistical power and cloud efficacy signals. Accordingly, immunotherapy in PTCL should be regarded as a field in early translation—with considerable promise but presently limited by incomplete biological insight and fragmented clinical evidence.

As the field progresses, genuine progress will require sophisticated frameworks that integrate multi-omic and spatial profiling to resolve the interplay between tumor-intrinsic drivers and the immune milieu. Such multi-dimensional analyses will be essential to systemically identify actionable immune vulnerabilities and redefine disease classification beyond the status quo histologic and lineage-based criteria. Ultimately, these advances may enable a shift from empirical immune modulation to biomarker-directed intervention, transforming immunotherapy in PTCLs from a theoretical concept into a precision discipline.

## 6. Conclusions

The therapeutic landscape of PTCLs is at an inflection point. While recent clinical trials demonstrate that immunotherapies can achieve responses in otherwise refractory disease, their efficacy is inconsistent and seldom durable. Current personalized strategies—such as PD-L1 immunohistochemistry, next-generation sequencing, or TRBC-restricted targeting—represent early steps toward a precision framework but require further validation and broader clinical application. The breadth of potential biomarkers reviewed herein attests to their potential utility in shaping the next generation of immunotherapy trials, yet their largely exploratory and poorly validated status highlights the parallel need for better validation and clinical integration in PTCL. Emerging technologies offer some promise to surmount many of the current limitations. Armored CAR-T cells engineered to secrete cytokines or resist checkpoint inhibition may overcome the issues of persistence and TME-mediated suppression. Allogeneic or gene-edited platforms could mitigate fratricide and reduce the risk of pan-T cell ablation, enabling safer and more scalable deployment. Bispecific antibodies and antibody-armed T cells may provide flexible, “off-the-shelf” alternatives that bypass manufacturing delays while still harnessing immune effectors in a controllable manner. Oncolytic viruses and immunomodulatory drugs add further opportunities to reshape the immune microenvironment, and biologically rational synergistic combinations of these modalities may ultimately generate deeper, more durable responses. Though the challenge posed by rare and heterogenous diseases like TCL is formidable, the next generation of trials must move beyond single-agent studies to adopt adaptive, biomarker-driven designs that incorporate ctDNA, immune-gene signatures, and TME characterization to directly test personalized strategies.

In summary, the future of PTCL therapy lies in a shift from empiricism toward precision. Achieving this will require coordinated efforts in translational research, adaptive trial design, and clinical implementation, but the growing complement of technologies outlined herein shows that such ambition will soon prove feasible. Personalized immunotherapy offers a compelling path forward for improving outcomes in this historically intractable disease.

## Data Availability

No new data were created or analyzed in this study. Data sharing is not applicable to this article.

## Abbreviations

The following abbreviations are used in this manuscript:

ACT	Adoptive cell transfer
ADCC	Antibody-directed cellular cytotoxicity
AITL	Angioimmunoblastic T cell lymphoma
ALCL	Anaplastic large cell lymphoma
ALK	Anaplastic lymphoma kinase
allo-HSCT	Allogeneic hematopoietic stem cell transplantation
AML	Acute myeloid leukemia
ATLL	Adult T cell leukemia/lymphoma
auSCT	Autologous stem cell transplantation
BIA-ALCL	Breast-implant-associated anaplastic large cell lymphoma
BV	Brentuximab vedotin
BV-CHP	Brentuximab vedotin, cyclophosphamide, doxorubicin and prednisolone
c-FLIP	cellular FLICE inhibitory protein
CAR	Chimeric Antigen Receptor
CHOP	Cyclophosphamide, adriamycin, vincristine, and prednisone
CPCT	Chidamide, prednisone, cyclophosphamide, and thalidomide
CR	Complete response
CRBN	Cereblon
CRR	Complete response rate
CRS	Cytokine release syndrome
CTCL	Cutaneous T cell lymphoma
ctDNA	Circulating tumor DNA
dMMR	Deficient mismatch repair
EATL	Enteropathy-associated T cell lymphoma
EBV	Epstein–Barr Virus
EFS	Event free survival
EGFR	Epidermal Growth Factor Receptor
ENKTL	Extranodal NK/T cell lymphoma
FDC	Follicular dendritic cell
GvHD	Graft-versus-host disease
HDACi	Histone deacetylase inhibitor
HEV	High endothelial venule
HIF	Hypoxia-Inducible Factor
HL	Hodgkin lymphoma
HSTCL	Hepatosplenic gamma delta T cell lymphoma
HTLV-1	Human T lymphotropic virus 1
ICANS	Immune cell-associated neurotoxicity syndrome
ICE	Innate cell engager
ICI	Immune checkpoint inhibitor
ICOSL	Inducible T cell costimulator ligand
IMIDs	Immunomodulatory imide drugs
KIR	Killer Immunoglobulin-like Receptor
mDC	Myeloid dendritic cell
MDSC	Myeloid-derived suppressor cell
MEITL	Monomorphic epitheliotropic intestinal T cell lymphoma
MHC	Major Histocompatibility Complex
MRD	Minimal residual disease
MSI	Microsatellite instability
nTFHL	Nodal T-follicular helper cell lymphoma
nTFHL-AI	Nodal T-follicular helper cell lymphoma, angioimmunoblastic type
NHL	Non-Hodgkin lymphoma
NK	Natural Killer Cell
ORR	Overall response rate
PCET	PD-1 antibody + chidamide + etoposide + thalidomide
PD	Progressive disease
PFS	Progression-free survival
PMBCL	Primary mediastinal B cell lymphoma
PR	Partial response
PTCL	Peripheral T cell lymphomas
PTCL-NOS	Peripheral T cell lymphoma, not otherwise specified
r/r	relapsed/refractory
sALCL	Systemic ALCL
SD	Stable disease
SIRPα	Signal regulatory protein alpha
SS	Sezary Syndrome
T-ALL	T cell acute lymphoblastic leukemia
T-LBL	T cell lymphoblastic lymphoma
TAM	Tumor-associated macrophage
TCL	T cell lymphoma
TCR	T cell receptor
Tfh	T-follicular helper
Th1	T-helper 1
Th17	T-helper 17
Th2	T-helper 2
TIL	Tumor-infiltrating lymphocyte
TMB	Tumor mutational burden
TME	Tumor Microenvironment
TRAIL	TNF-related apoptosis-inducing ligand
Treg	Regulatory T cell
TTNT	Time to next treatment
VEGF	Vascular endothelial growth factor
